# Sas3-mediated histone acetylation regulates effector gene activation in a fungal plant pathogen

**DOI:** 10.1128/mbio.01386-23

**Published:** 2023-08-29

**Authors:** Marta Suarez-Fernandez, Rocio Álvarez-Aragón, Ana Pastor-Mediavilla, Alejandro Maestre-Guillén, Ivan del Olmo, Agustina De Francesco, Lukas Meile, Andrea Sánchez-Vallet

**Affiliations:** 1 Centro de Biotecnología y Genómica de Plantas (CBGP, UPM-INIA), Universidad Politécnica de Madrid (UPM)—Instituto Nacional de Investigación y Tecnología Agraria y Alimentaria (INIA)/Consejo Superior de Investigaciones Científicas (CSIC), Madrid, Spain; 2 Department of Marine Sciences and Applied Biology, University of Alicante, Alicante, Spain; Universidad de Cordoba, Cordoba, Spain

**Keywords:** histone acetylation, *Zymoseptoria tritici*, Sas3, effector gene activation, chromatin remodeling, wheat

## Abstract

Effector proteins are secreted by plant pathogens to enable host colonization. Typically, effector genes are tightly regulated, have very low expression levels in axenic conditions, and are strongly induced during host colonization. Chromatin remodeling contributes to the activation of effector genes *in planta* by still poorly known mechanisms. In this work, we investigated the role of histone acetylation in effector gene derepression in plant pathogens. We used *Zymoseptoria tritici*, a major pathogen of wheat, as a model to determine the role of lysine acetyltransferases (KATs) in plant infection. We showed that effector gene activation is associated with chromatin remodeling, featuring increased acetylation levels of histone H3 lysine 9 (H3K9) and 14 (H3K14) in effector loci. We functionally characterized the role of *Z. tritici* KATs and demonstrated their distinct contributions to growth, development, and infection. Sas3 is required for host colonization and pycnidia production and is involved in the acetylation of H3K9 and H3K14 in effector loci and, consequently, in effector gene activation during plant infection. We propose that Sas3-mediated histone acetylation is required for the spatiotemporal activation of effector genes and the virulence of *Z. tritici*.

**IMPORTANCE:**

Pathogen infections require the production of effectors that enable host colonization. Effectors have diverse functions and are only expressed at certain stages of the infection cycle. Thus, effector genes are tightly regulated by several mechanisms, including chromatin remodeling. Here, we investigate the role of histone acetylation in effector gene activation in the fungal wheat pathogen *Zymoseptoria tritici*. We demonstrate that lysine acetyltransferases (KATs) are essential for the spatiotemporal regulation of effector genes. We show that the KAT Sas3 is involved in leaf symptom development and pycnidia formation. Importantly, our results indicate that Sas3 controls histone acetylation of effector loci and is a regulator of effector gene activation during stomatal penetration. Overall, our work demonstrates the key role of histone acetylation in regulating gene expression associated with plant infection.

## INTRODUCTION

Plant pathogens produce and secrete effectors into host tissues to facilitate colonization. Effectors have several functions, including suppression of the immune response, alteration of plant development, acquisition of nutrients, and interference with the host microbiota (1). Effectors are frequently required at specific phases of the infection cycle (2). Consequently, the transcriptional control of effector genes is key to providing the pathogen with a dynamic infection machinery. Despite the importance of tight effector gene regulation in fungal plant pathogens, the mechanisms involved remain mostly enigmatic.

Chromatin remodeling is a pivotal mechanism of gene regulation and involves post-translational modifications of histone tails, such as acetylation and methylation. These modifications provide a conserved mechanism that modulates the accessibility of the transcription machinery to the DNA and thereby alters gene expression (3 – 5). Writing enzymes, including methylases and acetylases, and erasing enzymes, such as demethylases and deacetylases, are dynamically involved in post-transcriptional modifications of histone tails in eukaryotes (6 – 8). Effector genes are frequently located in heterochromatic regions of the genome (8, 9). In plant-associated fungi, including *Leptosphaeria maculans*, *Epichloë festucae*, *Magnaporthe oryzae,* and *Z. tritici*, effector genes are enriched in trimethylation of histone H3 lysine 9 (H3K9) and/or 27 (H3K27) in the absence of the host (10 – 13). During plant colonization, effector gene activation is associated with a tightly regulated reduction in the methylation levels in H3K9 and/or H3K27, as shown in *E. festucae* and *Z. tritici*. Accordingly, disruption of the key enzymes involved in the methylation of H3K27 or H3K9 has been shown to enhance the expression of effector genes and secondary metabolite gene clusters (10 – 13). Thus, the derepression of effector genes during host colonization involves changes in the chromatin state.

Acetylation of specific residues of core histone tails has been shown to regulate transcription in eukaryotes (6, 7). Lysine acetyltransferases (KATs) transfer acetyl groups from acetyl-coenzyme A onto lysine residues of core histones and commonly form part of complexes (7). KATs are classified into different families, including the MYST (MOZ, YBF2/SAS3, SAS2, and TIP60), the GNAT (Gcn5-related *N*-acetyltransferase), the p300/CBP (protein of 300 kDa and CREB-binding protein) families, and the fungal-specific family Rtt109 (regulator of Ty1 transposition gene product 109) (7, 14, 15). Most KATs are part of multimeric complexes that harbor regulatory components that control KAT activity and substrate specificity (16). The Nua3 and Nua4 complexes contain Sas3 and Esa1, respectively, and acetylate histones H3 and H4 (17 – 19). Gcn5 is part of the SAGA and ADA complexes (20). Histone acetyltransferases in filamentous fungi have been reported to regulate several biological processes such as growth, reproduction, secondary metabolite synthesis, and pathogenicity. For instance, orthologs of Gcn5 mediate dimorphic changes, tolerance to stress, and virulence in *Ustilago maydis* (21); proline metabolism and secondary metabolite regulation in *Aspergillus nidulans* (22 – 24); and stress tolerance and conidiation in *Alternaria alternata* (25). KATs from the MYST family are involved in the growth and conidiation of *M. oryzae* and *A. alternata* (25, 26). In *Fusarium graminearum,* KATs from the GNAT and MYST families mediate secondary metabolite regulation and virulence (27). Histone acetylation is also involved in fungus-bacterium interactions. Upon interaction of the filamentous fungus *A. nidulans* with the bacterium *Streptomyces rapamycinicus*, fungal secondary metabolite gene clusters are induced. This process involves acetylation of H3K9 and acetylation of histone H3 at lysine 14 (H3K14), as well as Gcn5 protein activity (22). Likewise, we hypothesized that histone acetylation plays a major role in effector gene activation in fungus-plant interactions. Considering the important role of KATs in transcriptional activation in eukaryotes and given the fact that effector genes are derepressed during host colonization (7, 10, 28 – 30), we propose that an increase in histone acetylation levels regulates the activation of pathogen effector genes during plant infection.

*Z. tritici* is a major pathogen of wheat, causing significant yield losses in temperate climates (31). The infection cycle of *Z. tritici* initiates at the leaf surface with the germination of asexual or sexual spores. Emerged hyphae grow on the leaf surface and penetrate through the stomata. Subsequently, *Z. tritici* colonizes the apoplast and, after several days of infection, it forms asexual fruiting bodies known as pycnidia (32). Chlorotic and necrotic symptoms are only observed after several days of infection of the pathogen, prior to asexual reproduction (33). *Z. tritici* mainly grows as a filamentous fungus on the wheat leaf surface but can also grow as blastospores *in vitro* on rich media and occasionally on the leaf surface (34). Various effector genes are strongly induced during plant infection at different stages of the *Z. tritici* life cycle, *AvrStb6* and *Avr3D1* being activated during stomatal penetration and apoplast colonization. In contrast, the effector gene *Mycgr3G76589* (encoding a secreted cellulase from the family GH45; *ZtCel45A*) is expressed at later stages of the infection (35 – 38). Our integrative study aimed to determine the role of histone acetylation and KATs in effector gene regulation in *Z. tritici*. We demonstrated that dynamic histone acetylation of H3K9 and H3K14 is associated with expression activation of effector genes and host colonization.

## RESULTS

### *Z. tritici* has 7 KAT orthologs

We first aimed to identify KAT orthologs in *Z. tritici* by performing a BLAST search on annotated *Z. tritici* genes using previously characterized *Saccharomyces cerevisiae* KATs as queries. In addition, we used the dbHiMo database (39), which comprises histone-modifying enzymes from several fungal species including *Z. tritici*. A reverse BLAST analysis with the identified putative *Z. tritici* KAT orthologs was subsequently performed on the *S. cerevisiae* genome. We found three KAT orthologs from the MYST family, two from the GNAT family, and one from the fungal-specific family Rtt109 (Table 1). In addition, one ortholog of Gcn5-related *N*-acetyltransferase (Ngs1; Table 1) previously identified in *Candida albicans* was also identified in *Z. tritici* (40).

**TABLE 1:** Classification of lysine acetyltransferase (KAT) orthologs in *Zymoseptoria tritici* strain ST99CH_3D7

KAT nomenclature	Previous nomenclature	KAT family	Gene ID in *Z*. *tritici* strain ST99CH_3D7
KAT1	Hat1	GNAT	Not found
KAT2	Gcn5	GNAT	3D7.g4775
KAT5	Esa1	MYST	3D7.g9281
KAT6	Sas3	MYST	3D7.g4263
KAT8	Sas2	MYST	3D7.g7031
KAT9	Elp3	GNAT	3D7.g8500
KAT11	Rtt109	RTT109	3D7.g8867
-	Ngs1	GNAT	3D7.g2851

**Fig 1:**
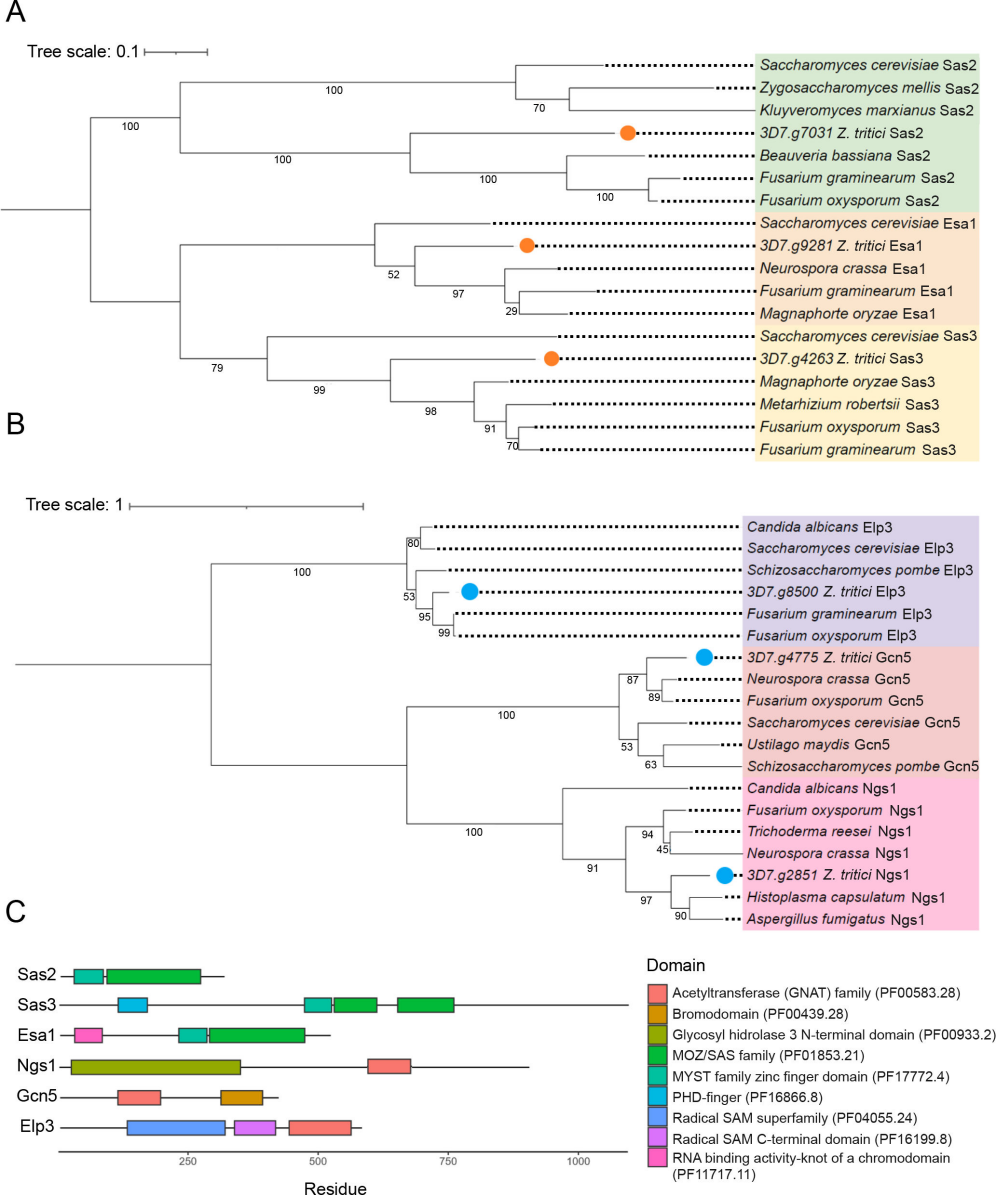
*Zymoseptoria tritici* has three lysine acetyltransferase (KAT) orthologs belonging to the MYST family (Esa1, Sas2, and Sas3) and three belonging to the GNAT family (Ngs1, Gcn5, and Elp3). (**A**) Phylogenetic tree of the MYST family protein members from different fungal organisms. MYST sequences belonging to *Z. tritici* are indicated with orange dots. The protein names are colored according to their classification: Sas2 (KAT8; green), Esa1 (KAT5; orange), and Sas3 (KAT6; yellow). (**B**) Phylogenetic tree of GNAT family proteins from different fungal organisms. GNAT sequences belonging to *Z. tritici* are indicated with blue dots. The protein names are colored according to the type of enzyme: Elp3 (KAT9; purple), Gcn5 (KAT2; light red), and Ngs1 (pink). The numbers below the branches represent the support values from 1,000 bootstrap replicates using the maximum likelihood method. Trees have been rooted using the midpoint root method. Tree scale indicates branch length in the tree. Units are given in residue substitution per site. (**C**) Domains identified in the KAT proteins of *Z. tritici*.

The BLASTp (Table S1) and dbHiMo analyses yielded a consistent classification of 3D7.g7031 as Sas2 (KAT8) and 3D7.g9281 as Esa1 (KAT5). However, the classification of 3D7.g4263 was conflicting since dbHiMo and BLASTp classified this protein as Esa1 and Sas3, respectively. Therefore, a phylogenetic tree was constructed using KATs of the MYST family from different fungal species. The phylogenetic analysis revealed that 3D7.g4263, 3D7.g7031, and 3D7.g9281 are orthologs of Sas3 (KAT6), Sas2 (KAT8), and Esa1 (KAT5), respectively (Fig. 1A; Table S2; Table 1). We identified the expected MOZ/SAS domain and the MYST family zinc finger domain in the *Z. tritici* orthologs of Esa1 and Sas2 using HMMER (Fig. 1C). In addition to these two domains, Esa1 harbors an RNA-binding domain near the N-terminus. Sas3 is the largest protein identified and contains two MOZ/SAS domains next to the MYST family zinc finger domain and a plant homeodomain (PHD) finger domain.

The phylogenetic analysis of KATs of the GNAT family indicated that 3D7.g2851, 3D7.g4775, and 3D7.g8500 cluster with Ngs1, Gcn5 (KAT2), and Elp3 (KAT9) proteins, respectively (Fig. 1B; Table S2; Table 1). All the identified KAT orthologs belonging to the GNAT family contain a GNAT acetyltransferase domain. In addition, Gcn5 has a bromodomain in its C-terminal region, while the Elp3 ortholog contains two radical SAM domains. Ngs1 contains a glycosyl hydrolase family 3 (GH3) domain (Fig. 1C), as previously described for orthologs of this KAT in other organisms (40).

### KAT genes in *Z. tritici* are differentially expressed during plant infection

**Fig 2:**
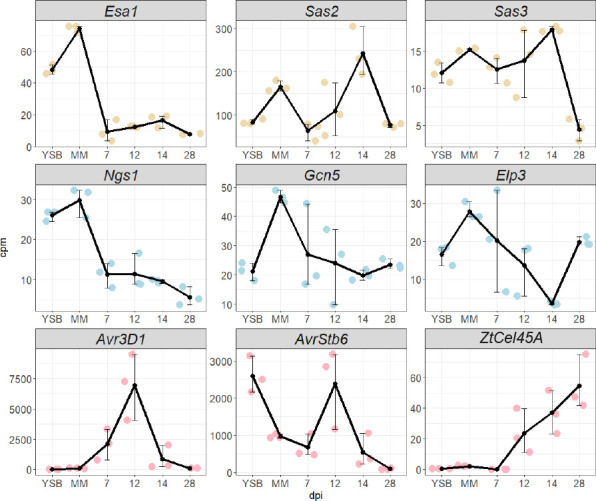
Specific regulation of *Zymoseptoria tritici* lysine acetyltransferase (KAT) genes during infection. Expression levels of the KAT genes *Esa1* (MYST family), *Sas2* (MYST family), *Sas3* (MYST family), *Ngs1* (GNAT family), *Gcn5* (GNAT family), and *Elp3* (GNAT family) and the effector genes *Avr3D1*, *AvrStb6,* and *ZtCel45A* under axenic conditions in two media with different nutrient content: yeast extract sucrose broth (YSB) and minimal medium (MM); and during infection at different time points (7, 12, 14, and 28 days post infection, dpi). Data were obtained from previously published RNA-seq studies (NCBI accessions: SRA SRP152081 and SRP077418) (34, 41). cpm: counts per million mapped reads.

We hypothesized that KATs involved in effector gene regulation might be expressed during plant infection and they might exhibit a similar expression pattern as effector genes. Therefore, we performed an expression analysis of the genes encoding the identified KATs and compared them to the expression of three effector genes formerly shown to be epigenetically regulated (10): *Avr3D1* (35), *AvrStb6* (36), and *Mycgr3G76589* (*ZtCel45A*) (37). For this purpose, we used data from previously published RNA-seq studies (34, 41). The MYST family orthologs (*Esa1*, *Sas2,* and *Sas3*) were expressed during host colonization, displaying low expression levels at the beginning of the infection and a peak of expression at 12–14 days post-infection (dpi). The three GNAT family members were also expressed during host colonization, *Elp3* exhibiting the lowest expression levels at 14 dpi (Fig. 2). Based on the different gene expression patterns of the KAT members, we hypothesized that they might have distinct roles in growth, development, and virulence.

### Histone acetylation levels in effector loci increase during plant infection

**Fig 3:**
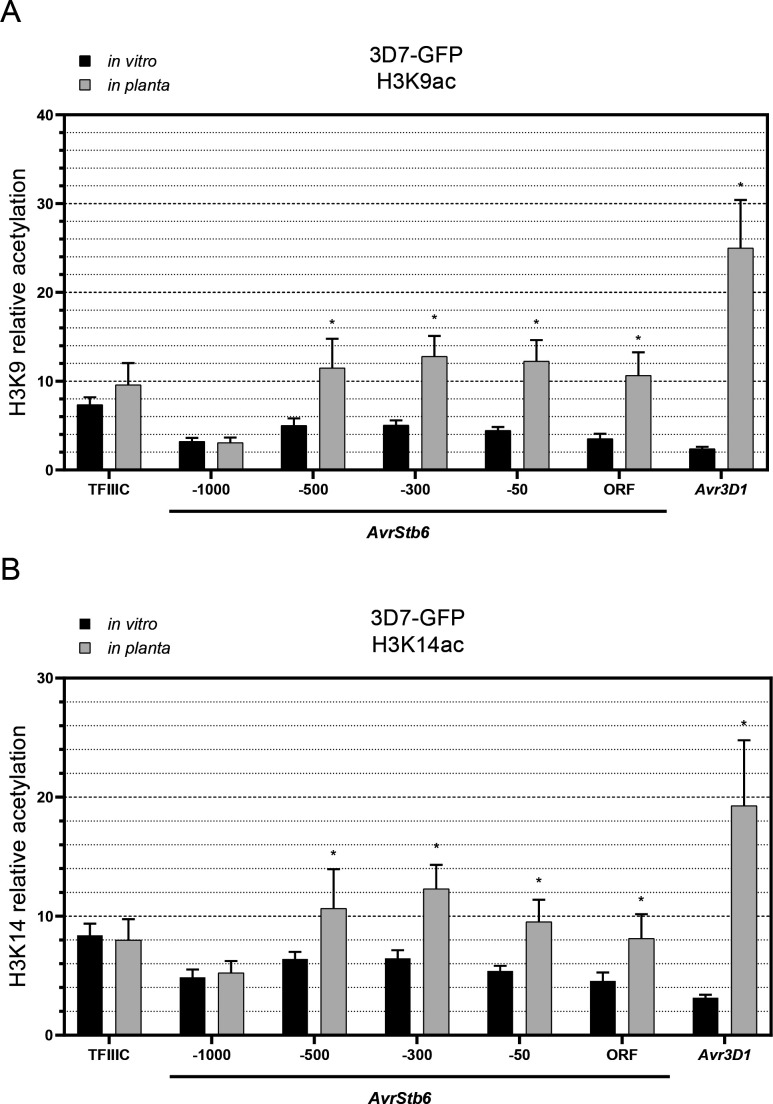
Acetylation levels of histone H3 lysine 9 (H3K9) and 14 (H3K14) in *Zymoseptoria tritici* increase during plant infection. Relative acetylation of H3K9 (**A**) and H3K14 (**B**) in 3D7-GFP *in vitro* and *in planta* in different regions of *AvrStb6*: −1,000 (from 1009 to 911 bp upstream of the start codon), −500 (from 532 to 444 bp upstream of the start codon), −300 (from 357 to 287 bp upstream of the start codon), −50 (from 94 to 20 bp upstream of the start codon), and ORF (from 131 to 212 downstream of the start codon); and approximately 300 bp (from 346 to 271 bp) upstream of the start codon of *Avr3D1. TFIIIC* (from 101 to 6 bp upstream of the start codon) was used as a control. Chromatin immunoprecipitation *in planta* was performed at 11 days post infection. Acetylation levels are shown relative to histone H3 levels. Bars show the average of three independent biological replicates and the error bars represent the standard error of the mean. Asterisks indicate significant differences between infection and axenic conditions according to two-way ANOVA and Bonferroni tests (*P < 0.05).

To evaluate the changes in acetylation of H3K9 and H3K14 during plant infection in effector loci, we performed a chromatin immunoprecipitation assay followed by quantitative PCR (ChIP-qPCR). We infected wheat plants with *Z. tritici* and harvested the second leaf at 11 dpi, which is approximately the time point at which maximum levels of effector transcripts can be observed [Fig. 2 (10)]. We additionally analyzed histone acetylation in *Z. tritici* grown under axenic conditions. We evaluated the acetylation levels of H3K9 and H3K14 in different regions upstream of *AvrStb6* (−1,000, –500, −300, and −50), and within the open reading frame (ORF) region. We also evaluated the acetylation of these two marks 300 bp upstream of the start codon of *Avr3D1*. A gene encoding the TFIIIC transcription factor complex unit (*3D7.g8520; TFIIIC*) was used as a control. As expected from previous work (42, 43), the acetylation levels at the region located approximately 1,000 base pairs (bp) upstream of the start codon of *AvrStb6* (−1,000) remained stable *in planta* compared to axenic conditions, similar to what we observed for the control *TFIIIC*. Remarkably, we detected an increase in the acetylation levels of H3K9 and H3K14 at the loci of *Avr3D1* and *AvrStb6* (Fig. 3). The ChIP-qPCR results support a possible role of histone acetylation in the activation of effector genes during infection.

### *Z. tritici* KAT orthologs are involved in growth and colony development under axenic conditions

To determine the function of the KAT orthologs of *Z. tritici*, we obtained loss-of-function mutants in the *Sas2*, *Sas3*, *Ngs1*, *Gcn5,* and *Elp3* genes. We first determined the role of the investigated KATs in development in the absence of the host. We measured the area of colonies of the KAT mutants grown on yeast-malt-sucrose agar (YMA; Fig. S1). Δ*Sas3* and Δ*Gcn5* colonies were significantly smaller than the colonies of the control. Interestingly, Δ*Sas2* lines displayed the opposite phenotype, with larger colony diameters than the controls, most likely due to their hyphal-like growth, as observed on the colony edges (Fig. S1). We therefore suggest that under axenic conditions, Sas2, Sas3, and Gcn5 might be involved in growth and/or development, with Sas2 probably being a negative regulator of growth and hyphal switching.

We addressed the role of the KATs in stress tolerance by assessing the performance of the mutants under different stresses, including high temperature (28°C), salt (NaCl; 0.5 M), H_2_O_2_ (1 mM), osmotic (sorbitol; 1 M), and cell wall (calcofluor white; 200 ng·μL^−1^, and Congo red; 2 mg·mL^−1^) stresses on YMA medium. In addition, we evaluated the growth of the KAT mutants on different carbon sources, such as fructose (5 g·L^−1^), galactose (50 mM), *N*-acetylglucosamine (GlcNAc, 2.5 mM), and glucose (2.5 mM) in a nutrient-poor minimal medium (MM) (44). Δ*Sas3* colonies were smaller than those of the control line (3D7-GFP) under all the conditions, supporting the role of Sas3 in growth and/or development in the absence of the host. Δ*Sas2* and Δ*Elp3* mutants grew similarly to the control under all the stress conditions, and Δ*Gcn5* and Δ*Ngs1* were slightly more resistant to Congo red than the control line (Fig. S1). Therefore, we conclude that *Z. tritici* KATs are not positive regulators of stress tolerance under axenic conditions.

### Sas3, Gcn5, and Elp3 are involved in virulence and/or pycnidia production

**Fig 4:**
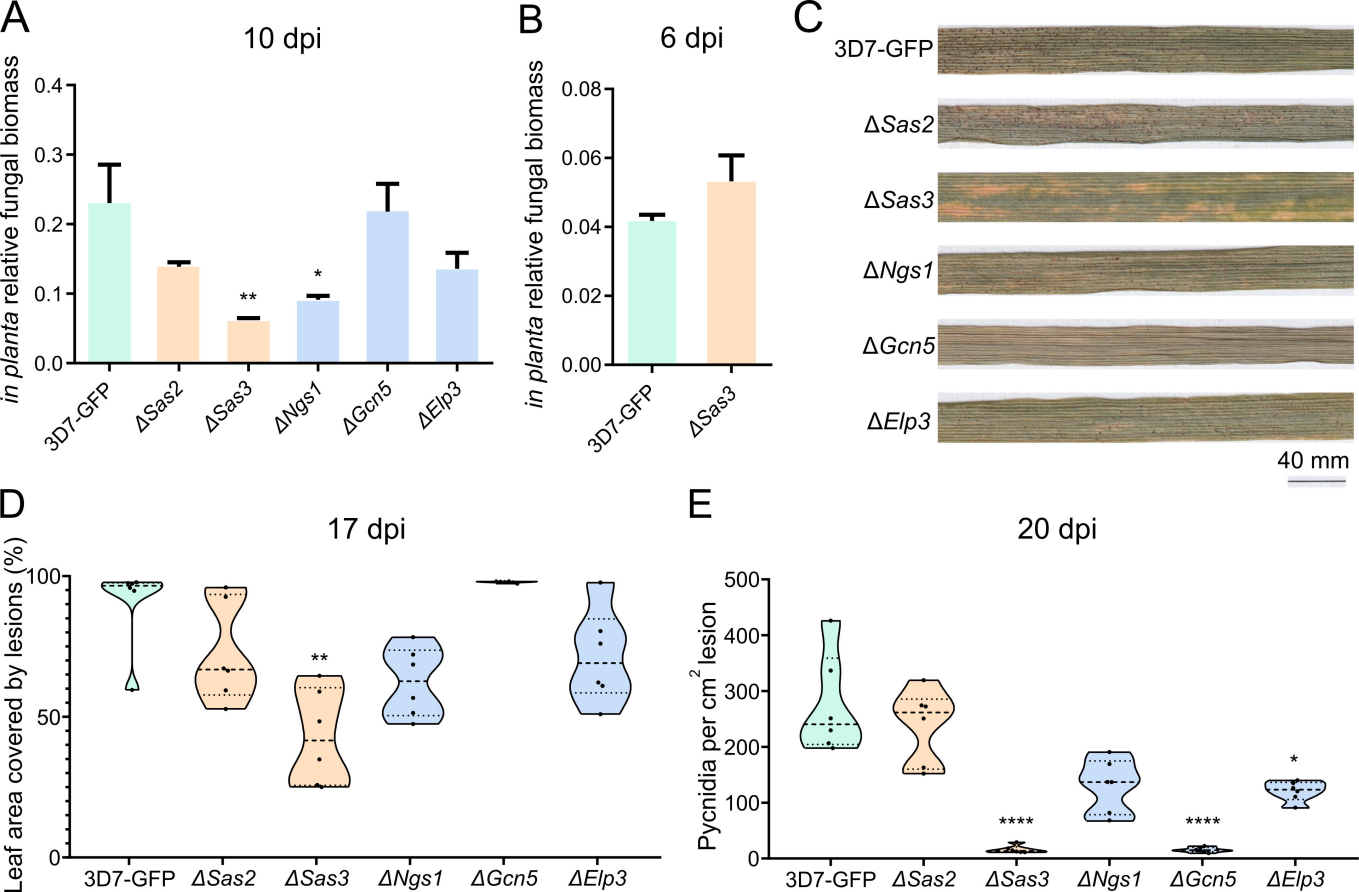
Lysine acetyltransferases (KATs) are involved in *Zymoseptoria tritici* infection. Relative fungal biomass of the control line (3D7-GFP) and the KAT mutants (Δ*Ngs1*, Δ*Sas2*, Δ*Sas3*, Δ*Gcn5,* and Δ*Elp3*) *in planta* at 10 days post infection (dpi) (**A**) and of 3D7-GFP and Δ*Sas3* at 6 dpi (**B**). Representative pictures of wheat leaves infected with 3D7-GFP and the knockout mutants in the KAT genes at 20 dpi (**C**). PLACL at 17 dpi (**D**) and pycnidia per cm^2^ of lesion at 20 dpi (**E**) of wheat plants infected with the control and the KAT mutants. In A, B, D and E, green represents 3D7-GFP, orange represents MYST family mutants and blue represents GNAT family mutants. Dashed lines represent the median, dotted lines represent first, and third quartiles and black dots represent individual data points. Asterisks indicate significant differences with 3D7-GFP according to Kruskal-Wallis and Dunn’s tests (*P < 0.05; **P < 0.01; ****P < 0.0001).

We investigated the role of KATs in host colonization on the susceptible wheat cultivar Runal (Fig. 4; Fig. S2 through S5). All the KAT mutants developed a similar *in planta* fungal biomass to that of the control at 10 dpi, except for Δ*Sas3* and Δ*Ngs1*, which exhibited lower biomass (Fig. 4A). However, at shorter time points (6 dpi), Δ*Sas3* grew to similar levels as the control (Fig. 4B; Fig. S3A). In accordance with the reduction in biomass, Δ*Sas3* developed fewer disease symptoms, as determined by the percentage of leaf area covered by lesions (PLACL), and fewer pycnidia (Fig. 4C through E; Fig. S2E through H and S4A and S5A and B). Infection by the Δ*Sas3* mutant produced orange spots on wheat leaves that were distinct from the symptoms produced by the control line (Fig. 4D; Fig. S4A). We observed a slightly faster production of symptoms by the Δ*Gcn5* mutant (Fig. 4D; Fig. S2M and N, S5A). Nevertheless, this faster spread of disease symptoms did not lead to increased fungal biomass or a higher production of pycnidia. Instead, we observed that Δ*Gcn5* generated very few pycnidia (Fig. 4E; Fig. S2O, P and S4A and S5B). Δ*Elp3* showed a slight reduction in symptom development (Fig. S5C) and in pycnidia density per lesion at 20 dpi (Fig. 4E; Fig. S2S and T, S5D), suggesting a contribution of Elp3 to asexual reproduction. In addition, the Δ*Sas2* mutant showed a slightly altered infection phenotype, manifested by orange spots on infected leaves, similar to the Δ*Sas3* phenotype, but without differences in PLACL or pycnidia production (Fig. 4; Fig. S2A through D, S4B). To confirm that the altered phenotypes of the mutants were due to the disruption of the KAT genes, we obtained complementation lines, which expressed the wild-type version of *Elp3*, *Gcn5,* and *Sas3* in the corresponding mutant backgrounds. We observed a restoration of virulence and pycnidia production in the Δ*Sas3*, Δ*Gcn5,* and Δ*Elp3* complementation lines (Fig. S4A and S5). These results demonstrate that Sas3 and Elp3 are involved in virulence and that Sas3 and Gcn5 mediate pycnidia formation, indicating the key role of *Z. tritici* KATs in virulence and reproduction.

### KAT mutants are impaired in expression regulation of effector genes under axenic conditions

**Fig 5:**
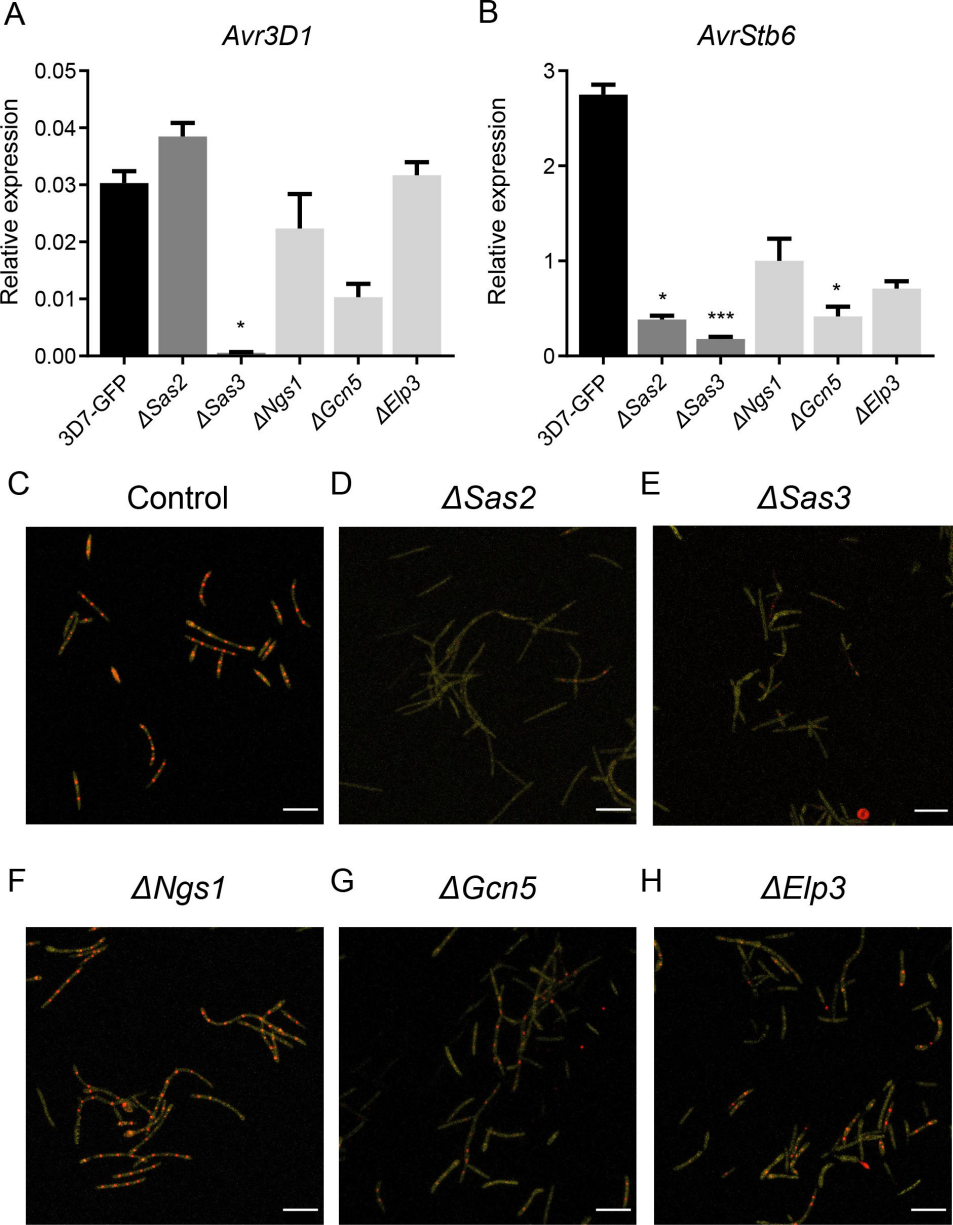
Lysine acetyltransferases (KATs) regulate effector gene expression under axenic conditions. Relative expression of the effector genes *Avr3D1* (**A**) and *AvrStb6* (**B**) in the control (3D7-GFP) and the *Zymoseptoria tritici* KAT mutants grown on yeast-malt-sucrose agar (YMA) for 6 d. *β-tubulin* and *histone H3* were used both as reference genes. Each bar corresponds to the mean expression value of three biological replicates and error bars represent the standard error of the mean. Asterisks indicate statistical differences with 3D7-GFP according to Kruskal-Wallis with uncorrected Dunn’s tests. (*P < 0.05; ***P < 0.001). Expression pattern of *AvrStb6* at the cellular level of *Z. tritici* lines grown for 6 d on YMA in the control reporter line (**C**); and the control reporter line lacking *Sas2* (**D**), *Sas3* (**E**), *Ngs1* (**F**), *Gcn5* (**G**), and *Elp3* (**H**). In the reporter line, *mCherry* fused to *histone 1* was expressed under the control of the *AvrStb6* promoter in the *AvrStb6* locus. This allowed the localization of the reporter to the nucleus (red dots) and therefore monitored the activity of the *AvrStb6* promoter at the single-cell level (10). Fungal blastospores are labeled with mTurquoise2 and are shown in yellow. Scale bars correspond to 25 µm.

We next determined the role of *Z. tritici* KATs in the regulation of the infection machinery by analyzing the expression levels of the effector genes *Avr3D1*, *AvrStb6,* and *ZtCel45A* by qRT-PCR under axenic conditions. The expression of *Avr3D1* was drastically reduced in Δ*Sas3* (Fig. 5A), and the expression of *AvrStb6* was reduced in Δ*Sas2,* Δ*Sas3,* and Δ*Gcn5* (Fig. 5B) under axenic conditions. *ZtCel45A* expression was unchanged in the mutants (Fig. S3D). To determine the role of the investigated KATs in effector gene regulation at the cellular level, we disrupted the KAT genes in a reporter line that harbors a histone 1 (*His1)-mCherry* fusion construct located at the *AvrStb6* locus, under the control of the *AvrStb6* promoter (10). The fusion of His1 with mCherry enabled its nuclear localization and allowed monitoring of *AvrStb6* expression at the cellular level. In the control reporter line growing under axenic conditions, mCherry was detected, indicating that the *AvrStb6* promoter is partially active when *Z. tritici* grows in the absence of the host (Fig. 5C and 2). Δ*Ngs1* and Δ*Elp3* showed the same expression pattern as the control (Fig. 5F and H), while Δ*Sas2*, Δ*Sas3,* and Δ*Gcn5* showed reduced mCherry accumulation under axenic conditions (Fig. 5D, E, and G). These results confirmed that *Z. tritici* Sas2, Sas3, and Gcn5 are involved in effector gene regulation in the absence of the host.

### Effector gene expression is altered during plant infection in the KAT mutants

**Fig 6:**
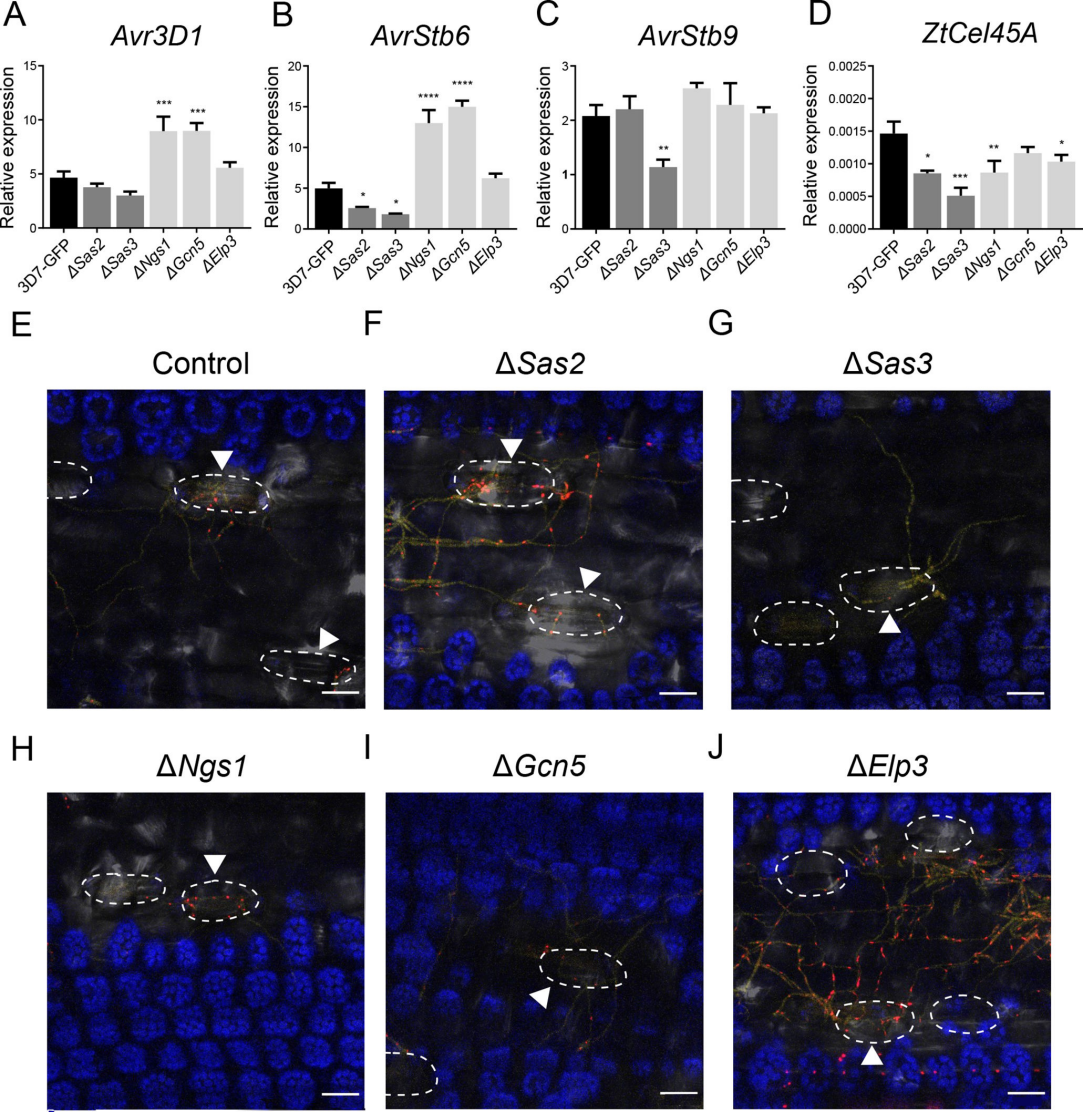
Lysine acetyltransefases (KATs) are involved in effector gene regulation during plant colonization. Relative expression of the effector genes *Avr3D1* (**A**), *AvrStb6* (**B**), *AvrStb9* (**C**), and *ZtCel45A* (**D**) in the control (3D7-GFP) and *Zymoseptoria tritici* KAT mutants (Δ*Ngs1*, Δ*Sas2*, Δ*Sas3*, Δ*Gcn5,* and Δ*Elp3*) during wheat infection at 10 days post infection (dpi), analyzed with qPCR. *β-tubulin* and *histone H3* were used both as reference genes. Bars correspond to the mean expression value of three biological replicates per treatment and error bars represent the standard error of the mean. Asterisks indicate significant differences with 3D7-GFP according to Kruskal-Wallis and Dunn’s tests (*P < 0.05; **P < 0.01; ***P < 0.001; ****P < 0.0001). Expression pattern of *AvrStb6* at the cellular level at 6 dpi in the (E) control reporter line; and the control reporter line lacking *Sas2* (**F**)*, Sas3* (**G**)*, Ngs1* (**H**)*, Gcn5* (**I**), and *Elp3* (**J**). In the reporter line, *mCherry* fused to *histone 1* was expressed under the control of the *AvrStb6* promoter in the *AvrStb6* locus. This allowed the localization of the reporter to the nucleus (red dots) and therefore monitored the activity of the *AvrStb6* promoter at the single-cell level (10). Fungal hyphae are labeled with mTurquoise2 and are shown in yellow. Chloroplasts are indicated in blue. White discontinuous lines indicate the stomata. Hyphae penetrating the stomata are indicated with an arrow. Scale bars correspond to 25 µm.

Effector genes are key for plant colonization and highly induced during infection (Fig. 2). We hypothesized that histone acetylation might be required for effector gene upregulation. We determined the expression levels of four effector genes [*Avr3D1*, *3D7.g7883* reannotated in (35)], *AvrStb6* (*3D7.g5586*), *AvrStb9* [*3D7.g741* (45)], and *ZtCel45A* (*Mycgr3G76589*/*3D7.g10118*) by qRT-PCR during plant infection in KAT knockout mutants (Fig. 6). The expression levels of *Avr3D1* and *AvrStb6* were higher in ∆*Ngs1* and ∆*Gcn5*, while *AvrStb6* expression was reduced in ∆*Sas2* and ∆*Sas3* at 10 dpi (Fig. 6A and B). A significant reduction in *AvrStb6* and *Avr3D1* expression was also shown in ∆*Sas3* at 6 dpi (Fig. S3B and C). We also observed reduced expression of *ZtCel45A* in all mutants except ∆*Gcn5* (Fig. 6D) and a reduction in *AvrStb9* expression in ∆*Sas3* (Fig. 6C). These results suggest that KATs are involved in infection and the proper regulation of effector gene expression during the early stages of plant colonization.

The above qRT-PCR analyses do not resolve expression levels in individual cells. The reporter line harboring the *mCherry* gene expressed under the control of the *AvrStb6* promoter and located in the *AvrStb6* locus allowed us to monitor the *AvrStb6* expression pattern at the cellular level. Although the qRT-PCR analyses were performed at 10 dpi, confocal microscopy pictures were taken at 6 dpi to avoid the autofluorescence produced by plants at later time points of the infection. The *AvrStb6* promoter showed little activity during hyphal growth on the plant surface but was strongly activated in hyphae approaching the stomata (Fig. 6D), as previously demonstrated (10). We investigated whether this expression pattern was mediated by KATs. Remarkably, all the analyzed mutants grew as hyphae on the leaf surface and reached leaf stomata. Interestingly, at 6 dpi, ∆*Sas3* showed only very low activation of the *AvrStb6* promoter even in hyphae attempting to penetrate the stomata (Fig. 6F). This confirms the previous qPCR-based observation of Sas3 being involved in effector gene regulation during infection (Fig. 5; Fig. S3). On the other hand, the activity of the *AvrStb6* promoter in ∆*Elp3* was higher than in the control, regardless of the proximity to stomata (Fig. 6I). ∆*Sas2*, ∆*Ngs1,* and ∆*Gcn5* displayed a similar *AvrStb6* expression pattern as the control at 6 dpi (Fig. 6E, G, and H). The reporter line revealed the contribution of KATs on effector gene regulation at the cellular level during penetration at 6 dpi (Fig. 6E through J). These results are complementary to the quantitative gene expression levels obtained with the global qPCR-based analysis at a later time point (10 dpi; Fig. 6A through D). The overall results demonstrate that Sas3 is involved in effector gene upregulation during stomata penetration in *Z. tritici*.

### Sas3 contributes to H3K9 and H3K14 acetylation of effector loci during plant infection

**Fig 7:**
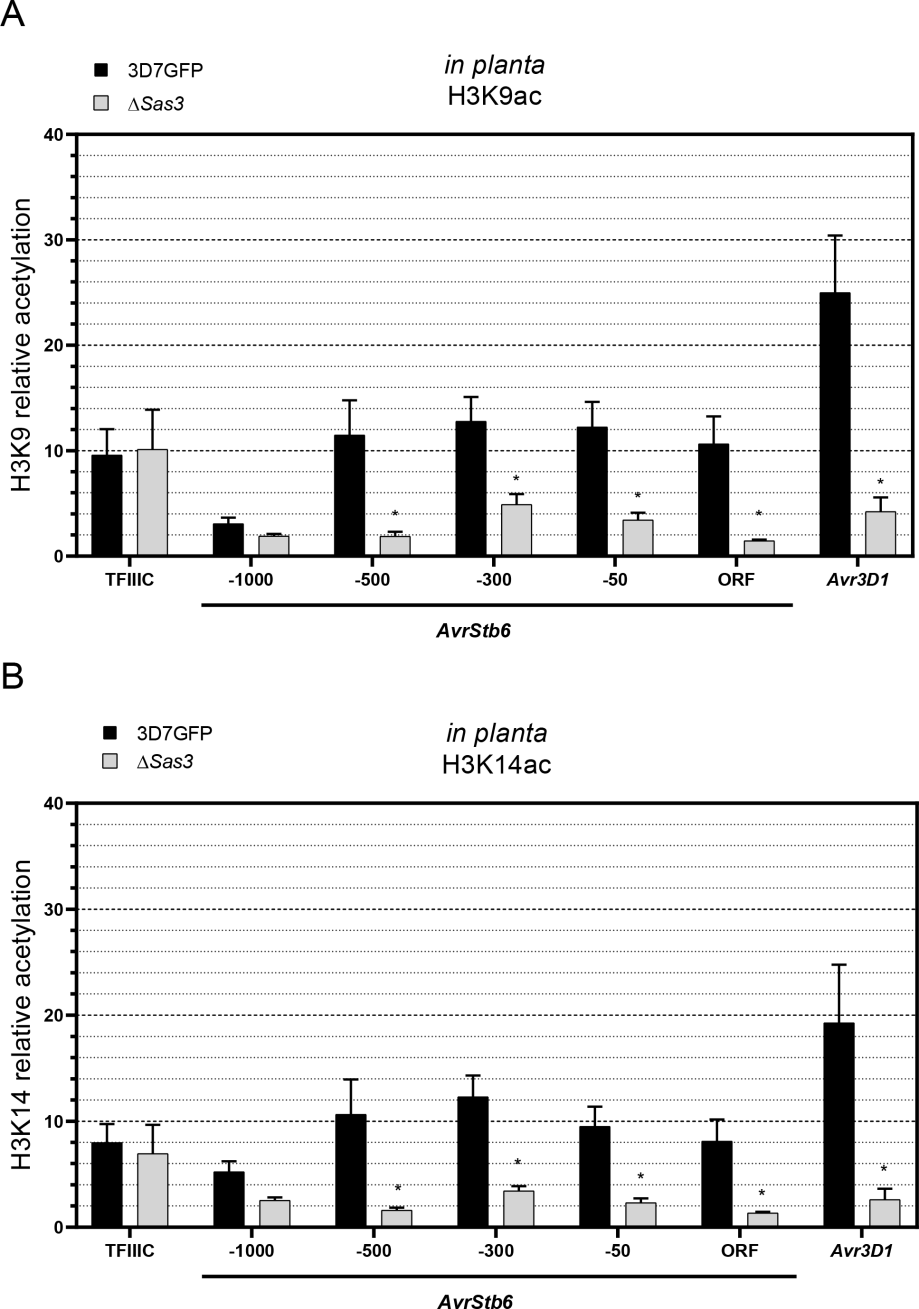
Histone H3 lysine 9 (H3K9) and 14 (H3K14) acetylation in *Zymoseptoria tritici* effector genes is mediated by Sas3 *in planta*. Relative acetylation of H3K9 (**A**) and H3K14 (**B**) in the control (3D7-GFP) and Δ*Sas3* in different regions of *AvrStb6*: −1,000 (from 1,009 to 911 bp upstream of the start codon), −500 (from 532 to 444 bp upstream of the start codon), −300 (from 357 to 287 bp upstream of the start codon), −50 (from 94 to 20 bp upstream of the start codon), and ORF (from 131 to 212 downstream of the start codon). We also evaluated the acetylation of these two marks approximately at 300 bp (from 346 to 271 bp) upstream of the start codon of *Avr3D1. TFIIIC* (from 101 to 6 bp upstream of the start codon) was used as a control. Chromatin immunoprecipitation *in planta* was performed at 11 days post infection. Acetylation levels are shown relative to histone H3 levels. Bars show the average of three independent biological replicates and the error bars represent the standard error of the mean. Asterisks indicate significant differences between Δ*Sas3* and 3D7-GFP according to two-way ANOVA and Bonferroni tests (*P < 0.05).

We subsequently evaluated whether Sas3-mediated expression regulation of effector genes is associated with histone acetylation during plant infection. We determined the acetylation levels of H3K9 and H3K14 during wheat infection in ∆*Sas3* lines. We observed a reduction in the relative acetylation levels of H3K9 (Fig. 7A) and H3K14 (Fig. 7B) in *AvrStb6* and *Avr3D1* in Δ*Sas3* compared to the control line during plant infection. As expected, acetylation levels of H3K9 and H3K14 in the control locus (*TFIIIC*) and 1,000 bp upstream of the start codon of *AvrStb6* were not affected by the *Sas3* deletion. These results demonstrate that Sas3 is involved in plant-associated acetylation of H3K9 and H3K14 in *AvrStb6* and *Avr3D1* effector loci. We showed that the reduced effector transcript levels and the hindered infection of ∆*Sas3* (Fig. 4 and 6) are associated with a reduction in histone acetylation in effector loci (Fig. 7).

## DISCUSSION

Exploring the mechanisms by which plant pathogens activate their infection machinery is key to understanding how the interaction between the host and the pathogen is established. In the past years, chromatin remodeling has been shown to be crucial for effector gene activation during plant infection (10, 11, 13, 30). However, the specific chromatin modifications that are involved in this activation remain largely unknown. In this work, we investigated the role of KATs and histone acetylation in the virulence of the fungal plant pathogen *Z. tritici*. We demonstrated for the first time that effector gene activation in a fungal pathogen is associated with histone acetylation during plant infection. We further demonstrated that Sas3-mediated histone acetylation dynamics mediate the upregulation of effector genes during plant infection.

A total of three KATs from the MYST family were identified in *Z. tritici*: Sas3, Sas2, and Esa1. We showed that Sas3 and Sas2 are involved in the regulation of well-characterized effector genes, including *AvrStb6*. Although Sas2 did not affect asexual reproduction and the speed at which necrotic lesions developed, it shaped the visual appearance of lesions, manifested by orange spots in lesions produced in infections by the *ΔSas2* mutant. We considered that this altered symptom development might be the result of misexpression of *AvrStb6* and potentially other effector genes in the *ΔSas2* mutant. Likewise, the Sas2 ortholog in *B. cinerea* (BcSas2) is involved in the regulation of virulence (46), suggesting a conserved role of Sas2 in effector gene activation in fungal pathogens. Disruption of *Sas3* in *Z. tritici* led to a reduction in virulence and pycnidia formation. Similarly, *Sas3* from *M. oryzae* was shown to be involved in virulence, as the deletion of this gene had a profound effect on fungal growth, development, asexual reproduction, germination, and appressorium formation (26). We additionally noticed that Sas3 is required for normal growth under axenic conditions since colony size was reduced in *ΔSas3*. Although this reduction in growth in the absence of the host could indirectly lead to a reduction in virulence, we suggest that Sas3 is directly involved in the regulation of virulence since (i) *ΔSas3* grows as hyphae on the leaf surface of wheat and is able to reach the stomata, (ii) *ΔSas3* biomass levels *in planta* are similar to those of the control at early stages of wheat infection (6 dpi), (iii) *AvrStb6* activation is impaired in the proximity to the stomata in *ΔSas3* mutants, and (iv) misactivation of effector genes occurs at stages of infection when the fungal biomass is similar to the control (6 dpi). Thus, we believe that the impaired virulence of *ΔSas3* mutants is most likely a consequence of effector gene misregulation, featuring reduced expression levels of *AvrStb6*, *AvrStb9,* and *ZtCel45A* during plant infection, highlighting the contribution of Sas3 in the activation of effector genes. Remarkably, mutants in Sas3 are impaired in histone acetylation of effector genes during plant infection, suggesting that Sas3-mediated acetylation is a crucial mechanism driving the transcriptional reprogramming of effector genes during plant infection in *Z. tritici*.

We identified three members of the GNAT KAT family in *Z. tritici*. Expression analysis of effector genes in GNAT mutants revealed that, in contrast to MYSTs, GNATs might be involved in negatively regulating effector genes, since higher levels of effector transcripts were detected in ∆*Gcn5* and ∆*Elp3*. This negative regulation is unlikely to be direct since acetylation typically leads to activation of gene expression. Therefore, we suggest that GNATs might not directly activate effector loci but they might indirectly regulate effector gene transcription through other transcriptional regulators. We suggest that in ∆*Gcn5* the higher expression levels of effector genes might lead to faster development of necrosis. This might be due to two possible scenarios. The high accumulation of effectors might lead to early recognition of the pathogen by the host, resulting in a strong immune response which might be manifested by cell death. Alternatively, misregulation of cell-death-inducing effectors might directly produce necrosis at earlier stages of the infection. In addition, we showed that Gcn5 is involved in asexual reproduction since the knockout mutant developed very few pycnidia. Interestingly, Gcn5 negatively regulates symptom development but positively regulates reproduction, supporting that different mechanisms govern virulence and pycnidia production, as previously shown (34).

H3K9 and H3K14 acetylation are well-known euchromatic marks (47). Accordingly, we observed an increase in histone acetylation levels in *Avr3D1* and *AvrStb6* during plant infection, along with the derepression of these two effector genes. We suggest that this increase in histone acetylation levels contributes to the *in planta*-specific upregulation of effector genes in *Z. tritici.* High *in planta-* H3K9 and H3K14 acetylation levels require a functional Sas3 in *Z. tritici*. It has been previously reported that the Sas3-containing complex NuA3 acetylates H3K14 but not H3K9 *in vitro* (16, 18). Thus, the reduction of H3K14 acetylation levels in ∆*Sas3* is likely a direct consequence of the loss of the activity of this KAT. Sas3 might also acetylate H3K9 *in vivo* in *Z. tritici* or the reduced levels of this mark in the knockout mutant might also be an indirect consequence of Sas3 loss of function. Genome-wide studies have shown that H3K9ac and H3K14ac co-occur in transcriptionally active sites (48), and it has been suggested that active histone modifications act coordinately to lead to transcription activation (16, 47). We propose that the lack of Sas3 activity alters the levels of H3K14 acetylation, which might affect the activity of other KATs, resulting in the reduction of H3K9 acetylation. This has been previously reported for other histone modifications (49, 50). Alternatively, it has been reported that transcriptional activity itself modulates the levels of histone acetylation (47). Therefore, we cannot discard that in ∆*Sas3*, a reduction in the transcription of the effector genes might lead to a reduction in the acetylation of H3K9. Previously, we reported a decrease in the levels of H3K27 and H3K9 trimethylation in effector loci during plant infection associated with effector gene derepression (10). We consider that this reduction of histone repressive marks and an increase in activating marks, such as those described in the current work, promote a local switch from repressive to permissive chromatin, allowing the access of nucleosome remodeling complexes and structural modifications in chromatin, including decondensation of the chromatin fiber. For the investigated effector genes, such changes in chromatin structure are very local and do not affect neighboring loci (10), suggesting the specific targeting of KATs to effector loci during plant infection. Substrate specificities have been reported to be mediated by certain subunits from KAT complexes or KAT domains that interact with nucleosomes (28). In Sas3 from *Z. tritici,* a PHD-finger domain was identified, which has a potential role in substrate specificity or interaction with regulatory proteins (51). In addition, the concerted expression of effector genes during plant infection most likely requires transcription factor activities, as demonstrated for the Zn_2_Cys_6_ family member transcription factor Pf2 from *L. maculans*. In this case, the coordinated action of trimethylation of H3K9 and Pf2 governs the specific expression pattern of effector genes (52). Accordingly, we propose that chromatin modifications and still unknown transcription factors might jointly act as derepressors of effector genes of *Z. tritici* during plant infection.

We have shown that histone modifications, involving acetylation and demethylation (10), mediate the activation of effector genes during plant infection. Elucidating the crosstalk between histone modifications, their direct or indirect function in effector gene regulation, and the role of classic transcriptional activators and repressors will help us to further understand the molecular mechanisms linking chromatin and stage-specific transcriptional changes. Future work aiming to unveil global changes in histone acetylation and methylation patterns during plant infection will shed more light on the contribution of these histone marks to the regulation of the infection machinery.

## MATERIALS AND METHODS

### Fungal and bacterial strains

We used the *Z. tritici* Swiss strain ST99CH_3D7 [(53); abbreviated as 3D7]. All mutants were obtained either in 3D7 expressing the codon-optimized version of the enhanced green fluorescent protein (eGFP) [3D7-GFP (10, 54)], or in a mutant reporter line that expresses *mCherry* fused to His1 under the control of the *AvrStb6* promoter and located within the *AvrStb6* locus (10). Stellar *Escherichia coli* HST08 cells (Takara Bio, Japan) and the *Agrobacterium tumefaciens* strain AGL1 were used for cloning and *Z. tritici* transformation, respectively.

### Bioinformatic tools

To identify and classify the KAT orthologs from *Z. tritici*, we first used the basic local alignment search tool [BLAST (55)] from the National Centre for Biotechnology Information (NCBI) using the previously characterized KAT protein sequences from *S. cerevisiae* as queries (Table S1). Reverse BLAST was also performed to confirm that the identified protein sequences in *Z. tritici* were KAT orthologs. In parallel, we used the dbHiMo web-based data browser (39). We obtained a multiple sequence alignment of the KAT orthologs [MUSCLE (56)] and a phylogenetic tree. The protein sequences used for constructing the phylogenetic tree were obtained from NCBI (Table S2). Phylogenetic trees were constructed using the Molecular Evolutionary Genetics Analysis [MEGA-X 11 (57)] software, applying the maximum likelihood (ML) method with 1,000 non-parametric bootstraps as statistical support. Trees were rooted using the midpoint rooting method. We edited the trees using the Interactive Tree Of Life [iTOL (58)] software. In addition, we identified protein domains in *Z. tritici* KATs using HMMER (59) including all databases (Pfam, TIGRFAM, Gene3D, Superfamily, PIRSF, and TreeFam) and represented the different protein domains using the R package “ragp” (60).

### Generation of *Z. tritici* transformants

Plasmids for targeted gene deletion by homologous recombination were assembled using the In-Fusion HD Cloning Kit (Takara Bio, Japan). Briefly, the nourseothricin resistance gene PCR amplified from pES1-NAT-GFP (61) was flanked by homology arms of ca. 1 kb and inserted into the KpnI-SbfI-linearized acceptor plasmid pCGEN (62). Similarly, constructs for genetic complementation were generated by assembling gene sequences spanning from ca. 1 kb upstream of the start codon to right before the stop codon and the C-terminal 4xMyc-tag (63) into XhoI-linearized pLM1 plasmid (64). Primers are listed in Table S3. *Z. tritici* gene deletion and complementation mutants were obtained by *A. tumefaciens*-mediated transformation as previously described (10, 65) using nourseothricin (25 µg·mL^−1^) and hygromycin (100 µg·mL^−1^) for selection, respectively. Despite the presence of homology arms in the T-DNA, the transformation of *Z. tritici* typically yields high frequencies of ectopic insertions by non-homologous end-joining instead of or in addition to targeted insertions by homologous recombination. To distinguish deletion mutants from ectopic insertion mutants, a PCR-based mutant screening was performed using either purified genomic DNA as a template or directly adding liquid culture to the PCR. The screening method includes a primer binding site present between the nourseothricin resistance gene and the downstream homology arm. The sequence of this screening primer binding site was chosen to match the sequence of the gene to be deleted in a way that yields two distinct amplicons in the deletion and ectopic insertion mutants when combined with a primer binding site located in the region downstream of the homology arm (Fig. S6). Since this screening method yields distinct amplicons for both deletion and ectopic insertion mutants, failed PCRs can easily be identified by the lack of both amplicons. Furthermore, the presence of both amplicons in the same reaction allows the identification of impure mutant lines and heterokaryons. Insertion copy numbers were determined by qPCR and mutant lines with multiple inserts were discarded. At least two independent lines were obtained per mutant and used for subsequent experiments.

### Infection assays

Infection assays were performed on wheat (*Triticum aestivum* L.) plants of cultivar Runal grown for 15 d at 18°C during the day and 15°C during the night, with 16 h of light and 65% of relative humidity. In total, 16 seeds of cultivar Runal were sown in 11 × 11 × 12 cm pots with a peat-based substrate. Plants were fertilized after 1 week (Universal fertilizer, COMPO, Münster, Germany). The fungal inoculum was prepared 1 week before the infection by inoculating 50–100 μL of glycerol stock in 50 mL of yeast extract-peptone-dextrose broth (YPD; yeast extract 10 g·L^−1^, peptone 20 g·L^−1^, and dextrose 20 g·L^−1^) amended with kanamycin (50 µg·mL^−1^). Spore suspensions were prepared as described (35) and quantified using a BLAUBRAND bright-line Neubauer improved hemocytometer (0.100 mm depth, 0.0025 mm^2^ area; Brand, Wertheim, Germany), except for the ChIP-qPCR experiment in which we used the Spore Counter macro vs 2.13 (https://github.com/jalassim/SporeCounter.git; Julien Alassimone; ETH-Zürich, Switzerland).

Wheat infection assays were performed using fungal suspensions at a concentration of 10^7^ spores·mL^−1^ in 0.1% Tween-20, as previously described (35). Each pot was sprayed either with 12.5 mL 0.1% Tween-20 for mock treatment or with 12.5 mL spore suspension for controls and mutants. At least two independent mutant lines were used to evaluate symptom development and pycnidia production. Symptoms produced by *Z. tritici* were analyzed on the second leaf at two different time points using ImageJ (66) and an automated image analysis method (67). The PLACL and pycnidia density (pycnidia counts per square centimeter of lesion) were used as a proxy for virulence and asexual reproduction, respectively.

### Developmental assays under axenic conditions

We performed fitness assays with *Z. tritici* mutants in the absence of the host. A 3 µL drop of *Z. tritici* spore suspensions at 10^6^, 10^5^, 10^4,^, and 10^3^ spores·mL^−1^ was placed on different types of media: YMA, YMA supplemented with NaCl (0.5 M), H_2_O_2_ (1 mM), sorbitol (1 M), calcofluor white (200 ng·μL^−1^), or Congo red (2 mg·mL^−1^), MM [Voguel’s medium (44)], and MM supplemented with fructose (5 g·L^−1^), galactose (50 mM), GlcNAc (2.5 mM), or glucose (2.5 mM). Inoculated agar plates were incubated at 18°C. An additional plate of YMA was incubated at 28°C. Pictures were taken after 6 d. The area of individual colonies of the mutants under axenic conditions was estimated by inoculating ca. 100 colony forming units on YMA. Three independent replicates of each mutant were performed. After 8 d of incubation at 18°C, pictures of the plates were taken, and the colony size was analyzed using ImageJ.

### Confocal laser scanning microscopy assays

Confocal assays were performed on a Zeiss LSM 880 super-resolution confocal microscope with fast Airyscan. The emission settings were as follows: 511– 564 nm for the eGFP channel, 603–623 nm for the mCherry channel, 460–480 nm for the mTurquoise2 channel, and 692–697 nm for the chloroplast detection. For excitation, an argon (488 nm) laser was used for track 1 (mCherry and chloroplasts), and a diode laser (405 nm) was used for track 2 (mTurquiose2). The bright field is also shown. Image processing was performed using Fiji (68) and included the generation of maximum intensity Z-projections for merging channels and the addition of calibration bars. Colors were selected manually to facilitate channel identification. At least two independent lines per transformant were used. Experiments were performed at least twice.

### Effector gene expression analysis

Axenically grown cultures obtained as for the infection assays were filtered through a nylon membrane and centrifuged at 5,000 × *g*, 4°C for 5 min. The supernatant was discarded and fungal pellets were ground in liquid N_2_, using mortar and pestle. Infected plant tissue was collected at 6 and 10 dpi. The second leaves of 8 cm (after discarding 2 cm from the tip) were used for RNA extraction. Each replicate consisted of two leaves. At least three biological replicates were analyzed per treatment. RNA was extracted with Trizol (Life Technologies), purified (RNAeasy Mini Kit, QIAGEN Inc., The Netherlands), and treated with DNAse (QIAGEN Inc., The Netherlands). cDNA was synthesized using the Primescript RT reagent kit (Takara Bio, Japan). qPCRs were performed in a LightCycler480 II (Roche Diagnostics International AG, Rotkreuz, Switzerland) using the primers listed in Table S3, and the data were analyzed with the LightCycler 480 software (Roche Diagnostics International AG, Rotkreuz, Switzerland) using *histone H3* (*3D7.g6784*) and *β-tubulin* (*3D7.g2064*) as reference genes.

### Fungal biomass quantification *in planta*

Relative fungal biomass *in planta* was estimated by RT-qPCR, as described in the section above. We used plants infected with the control and the mutants at 6 and 10 dpi. We calculated the relative biomass of each fungal genotype using the fungal housekeeping genes *histone H3* and *β-tubulin*, and the plant housekeeping gene *TaCDC48* (*T. aestivum* cell division control protein 48 homolog E-like; *Traes4A02G035500*; Table S3) (69). Three biological replicates were analyzed per treatment. Each replicate consisted of 2 leaf sections of 8 cm (in which 2 cm from the tip were discarded).

### Chromatin extraction and immunoprecipitation

For *in vitro* assays, *Z. tritici* strain 3D7 was grown on YMS (0.4% yeast extract, 0.4% malt extract, and 0.4% sucrose). For *in vitro* chromatin extraction, 150 mg of tissue was used, while for *in planta* chromatin extraction 250 mg of tissue was used. The micrococcal nuclease (M0247S; New England Biolabs, Ipswich, MA, USA) reaction was performed at 37°C for 20 min. Chromatin fixation, immunoprecipitation, and de-crosslinking were performed as previously described (9, 10). Antibodies such as anti-H3 (ab1791, Abcam, Cambridge, UK), anti-H3K9ac (ab10812, Abcam, Cambridge, UK), and anti-H3K14ac (ab52946, Abcam, Cambridge, UK) were applied in 1:200 ratio. Subsequent qPCR was carried out on a LightCycler 480 instrument (Roche Diagnostics International AG, Rotkreuz, Switzerland) using the primers indicated in Table S3 and previously designed (10). The regions of *AvrStb6* analyzed are as follows: −1,000 (from 1,009 to 911 bp upstream of the start codon), −500 (from 532 to 444 bp upstream of the start codon), −300 (from 357 to 287 bp upstream of the start codon), −50 (from 94 to 20 bp upstream of the start codon), and ORF (from 131 to +212 downstream of the start codon). Acetylation levels were estimated as relative levels of H3K9ac and H3K14ac normalized to histone H3, as previously described (70, 71).

### Statistics

Statistical analysis and graphic representations were performed using either RStudio version 1.4.1717 (72) or GraphPad Prism 8.0.2 for Windows (GraphPad Software, San Diego, California). For conducting the statistical analyses, the Gaussian distribution of the data was tested using the Shapiro-Wilk normality test and the homogeneity of variances was analyzed using the Brown-Forsythe test. If the data followed a normal distribution and preserved homoscedasticity, the parametric ordinary one-way ANOVA test was applied together with Fisher’s Least Significant Difference (LSD) test (*P*-value < 0.05). If the aforementioned assumptions were not met, the non-parametric Kruskal-Wallis test was applied together with uncorrected Dunn’s test (*P*-value < 0.05). In the case of ChIP data, two-way ANOVA and Bonferroni analyses were performed (*P*-value < 0.05). All raw data used for performing main text and supplementary figures are available in the supplementary material.

## Data Availability

The authors declare that the raw data of all the experiments are included in the supplemental material (Raw Data).

## SUPPLEMENTAL MATERIAL

Figure S1 mbio.01386-23-s0001.pdf Sas3, Sas2 and Gcn5 regulate under axenic conditions growth and development of *Z. tritici*.

Figure S2 mbio.01386-23-s0002.pdf Infection assays of at least two independent mutant lines of lysine acetyltransferases (KATs) from the MYST and GNAT families.

Figure S3 mbio.01386-23-s0003.pdf Fungal biomass *in planta* is not affected in ∆*Sas3* and ∆*Gcn5* at early stages of the infection.

Figure S4 mbio.01386-23-s0004.png ∆*Sas3*, ∆*Gcn5*, and ∆*Sas2* produce anormal macroscopic disease symptoms.

Figure S5 mbio.01386-23-s0005.pdf Complementation of Δ*Sas3*, Δ*Gcn5*, and Δ*Elp3* recover the virulence phenotype.

Figure S6 mbio.01386-23-s0006.pdf PCR screening strategy to identify gene deletion mutants.

Data set 1 mbio.01386-23-s0007.xlsx Raw data of figures and supplemental material.

Table S1 mbio.01386-23-s0008.docx Five lysine acetyltransferases (KATs) from the MYST and GNAT families in *Z. tritici* were identified using BLASTp.

Table S2 mbio.01386-23-s0009.docx NCBI accession numbers used for performing the phylogenetic tree.

Table S3 mbio.01386-23-s0010.docx Primers used in this work.

ASM does not own the copyrights to Supplemental Material that may be linked to, or accessed through, an article. The authors have granted ASM a non-exclusive, world-wide license to publish the Supplemental Material files. Please contact the corresponding author directly for reuse.
