# Supplementary material for: Sas3-mediated histone acetylation regulates effector gene activation in a fungal plant pathogen
Source: mBio. 2023 Aug 29;14(5):e01386-23. doi: 10.1128/mbio.01386-23 (PMC10653901; doi:10.1128/mbio.01386-23)
Supplement: Figure S1 — Sas3, Sas2 and Gcn5 regulate under axenic conditions growth and development of Z. tritici. [file mbio.01386-23-s0001.pdf]

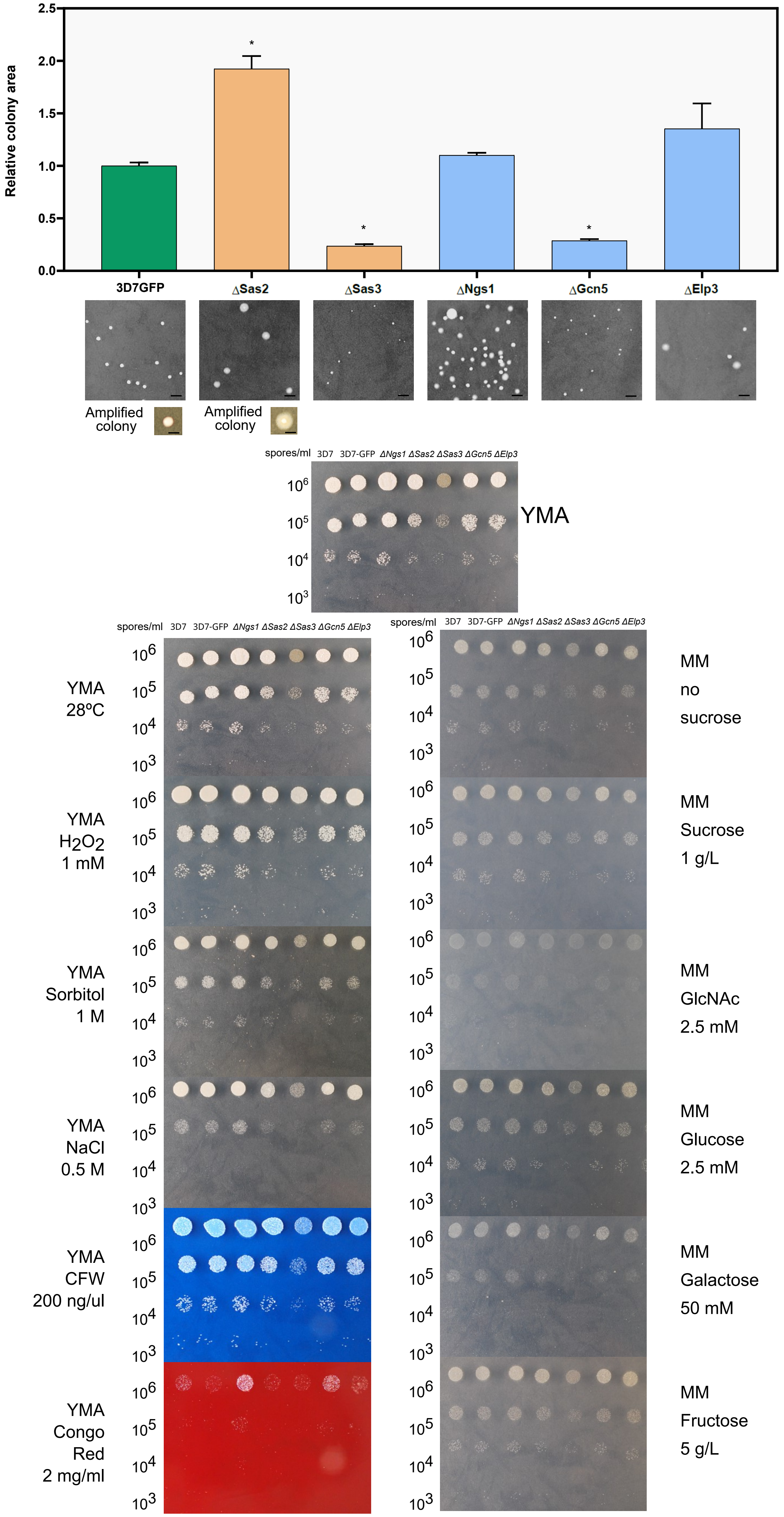

Figure S1. Sas3, Sas2 and Gcn5 regulate under axenic conditions growth and development of *Z. tritici*. Colony area of the Lysine acetyltransferase (KAT) mutants (ΔNgs1, ΔSas2, Δsas3, ΔGcn5 and ΔElp3) grown for 8 days on yeast-malt-sucrose agar (YMA) relative to the mean area of the colonies of the control (3D7-GFP). Green bar represents 3D7-GFP, orange bars represent MYST-family mutants and blue bars represent GNAT-family mutants. Bars represent the average of three independent biological replicates and error bars represent the standard error of the mean. Asterisks indicate significant differences with 3D7-GFP according to Kruskal-Wallis and uncorrected Dunn's tests ($p < 0.05$). A representative image of colonies of each mutant is shown. Additionally, an amplification of a colony of the control and ΔSas2 are shown. The scale bar corresponds to 3 mm. Three μL of fungal spore suspensions at a concentration of 106, 105, 104 and 103 spores·mL-1 of the controls (3D7 and 3D7-GFP), ΔNgs1, ΔSas2, ΔSas3, ΔGcn5 and ΔElp3 were inoculated. Media used were yeast-malt-sucrose agar (YMA); YMA supplemented with NaCl (0.5 M), H2O2 (1 mM), sorbitol (1 M), Calcofluor white (200 ng·μL-1) or Congo red (2 mg·mL-1); minimal medium (MM; Voguel's), MM supplemented with fructose (5 g·L-1), galactose (50 mM), GlcNAc (2.5 mM), or glucose (2.5 mM). Plates were incubated at 18°C for 6 days. One additional plate of YMA was incubated at 28°C.
