# Supplementary material for: Sas3-mediated histone acetylation regulates effector gene activation in a fungal plant pathogen
Source: mBio. 2023 Aug 29;14(5):e01386-23. doi: 10.1128/mbio.01386-23 (PMC10653901; doi:10.1128/mbio.01386-23)
Supplement: Figure S2 — Infection assays of at least two independent mutant lines of lysine acetyltransferases (KATs) from the MYST and GNAT families. [file mbio.01386-23-s0002.pdf]

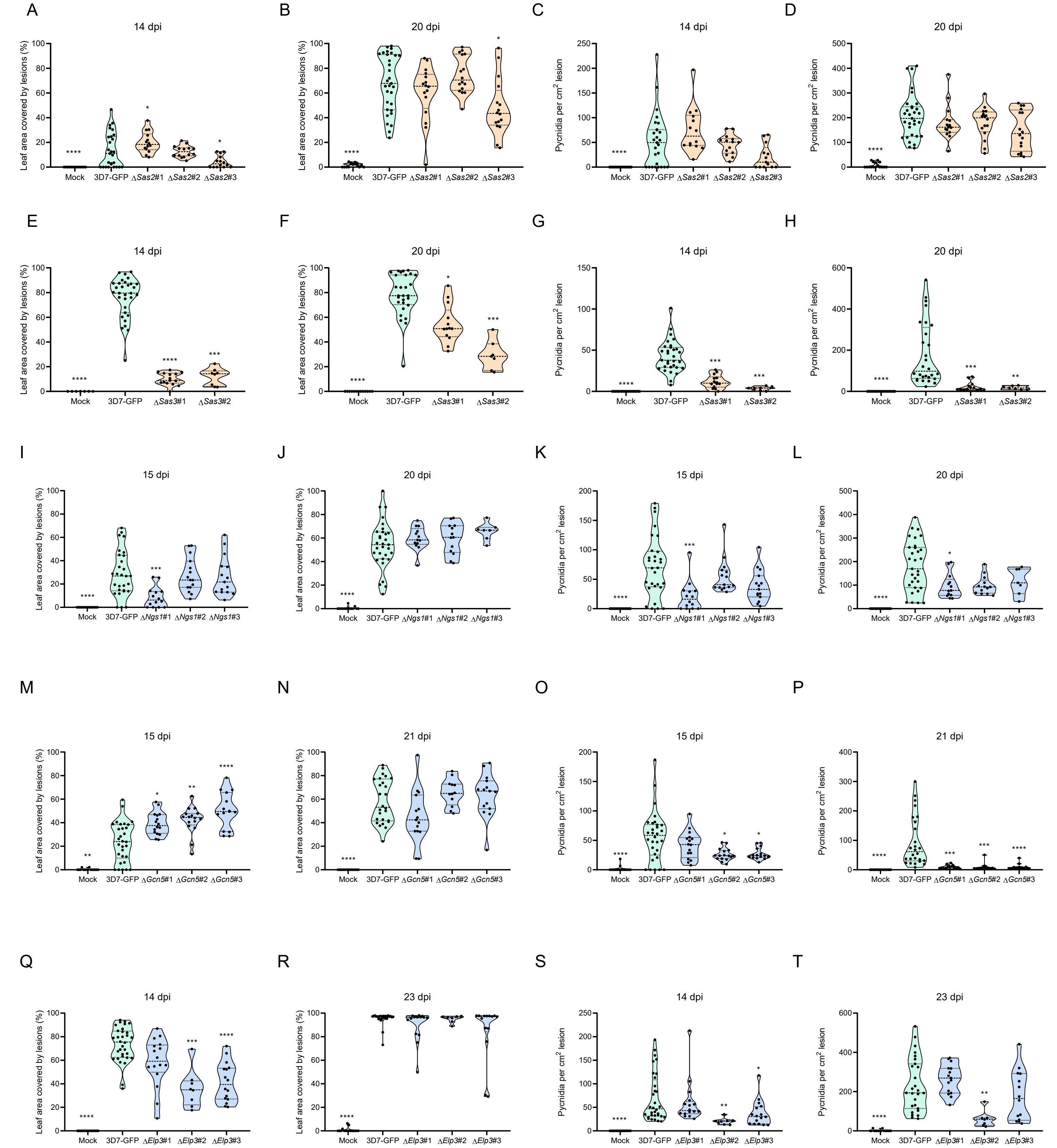

Figure S2. Infection assays of at least two independent mutant lines of lysine acetyltransferases (KATs) from the MYST and GNAT families. Percentage of leaf area covered by lesions (PLACL) at 14 (A) and 20 days post infection (dpi) (B) and pycnidia per cm^2 lesion at 14 (C) and 20 dpi (D) in the three independent ΔSas2 lines (#1, #2, #3). PLACL at 14 dpi (E) and 20 dpi (F) and pycnidia per cm^2 lesion at 14 dpi (G) and at 20 dpi (H) of two independ ent ΔSas3 mutant lines (#1, #2). PLACL at 15 (I) and 20 dpi (J) and pycnidia per cm^2 lesion at 15 (K) and 20 dpi (L) in three ΔNgs1 independent lines (#1, #2, #3). PLACL at 15 (M) and 21 dpi (N) and pycnidia per cm^2 lesion at 15 (O) and at 21 dpi (P) of three independent lines of ΔGcn5 (#1, #2, #3). PLACL at 14 (Q) and 23 dpi (R) and pycnidia per cm^2 lesion at 14 (S) and at 23 dpi (T) of three independent lines of ΔElp3 (#1, #2, #3). Dashed lines represent the median, dotted lines represent first, and third quartiles and black dots represent individual data points. Asterisks indicate statistically significant differences with the control (3D7-GFP) according to Kruskal-Wallis non-parametric statistical and posthoc uncorrected Dunn's tests (* $p < 0.05$; ** $p < 0.01$; *** $p < 0.001$; **** $p < 0.0001$).
