# Supplementary material for: Sas3-mediated histone acetylation regulates effector gene activation in a fungal plant pathogen
Source: mBio. 2023 Aug 29;14(5):e01386-23. doi: 10.1128/mbio.01386-23 (PMC10653901; doi:10.1128/mbio.01386-23)
Supplement: Figure S3 — Fungal biomass in planta is not affected in ∆Sas3 and ∆Gcn5 at early stages of the infection. [file mbio.01386-23-s0003.pdf]

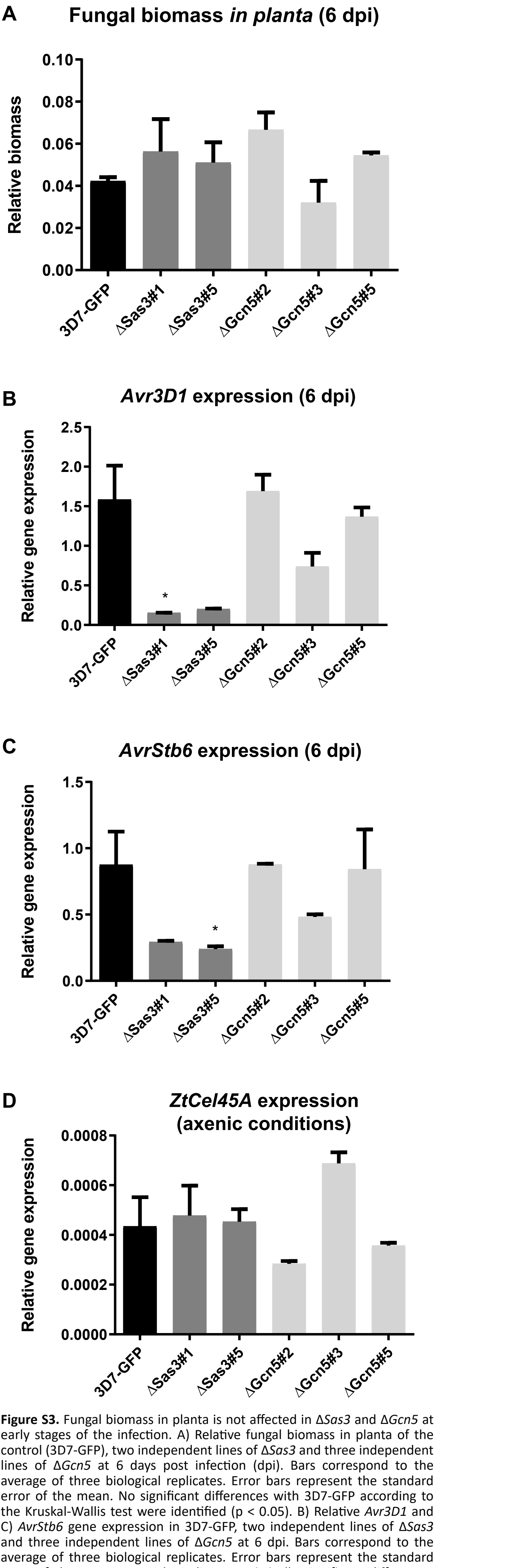

Figure S3. Fungal biomass in planta is not affected in Δ*Sas3* and Δ*Gcn5* at early stages of the infection. A) Relative fungal biomass in planta of the control (3D7-GFP), two independent lines of Δ*Sas3* and three independent lines of Δ*Gcn5* at 6 days post infection (dpi). Bars correspond to the average of three biological replicates. Error bars represent the standard error of the mean. No significant differences with 3D7-GFP according to the Kruskal-Wallis test were identified ($p < 0.05$). B) Relative *Avr3D1* and C) *AvrStb6* gene expression in 3D7-GFP, two independent lines of Δ*Sas3* and three independent lines of Δ*Gcn5* at 6 dpi. Bars correspond to the average of three biological replicates. Error bars represent the standard error of the mean. Asterisks indicate statistically significant differences with 3D7-GFP according to the Kruskal-Wallis test (* $p < 0.05$). D) The expression pattern of *ZtCel45A* is not altered in KAT mutants under axenic conditions. Relative expression of *ZtCel45A* in 3D7-GFP and the KAT mutants grown on yeast-malt-sucrose agar (YMA) for 6 days. *B-tubulin* and *Histone H3* were used as reference genes. Each bar corresponds to the average of 3 biological replicates. Error bars represent the standard error of the mean. No significant differences with 3D7-GFP according to the Kruskal-Wallis test were found ($p < 0.05$).
