# Supplementary figures and images for: Sas3-mediated histone acetylation regulates effector gene activation in a fungal plant pathogen
Source: mBio. 2023 Aug 29;14(5):e01386-23. doi: 10.1128/mbio.01386-23 (PMC10653901; doi:10.1128/mbio.01386-23)

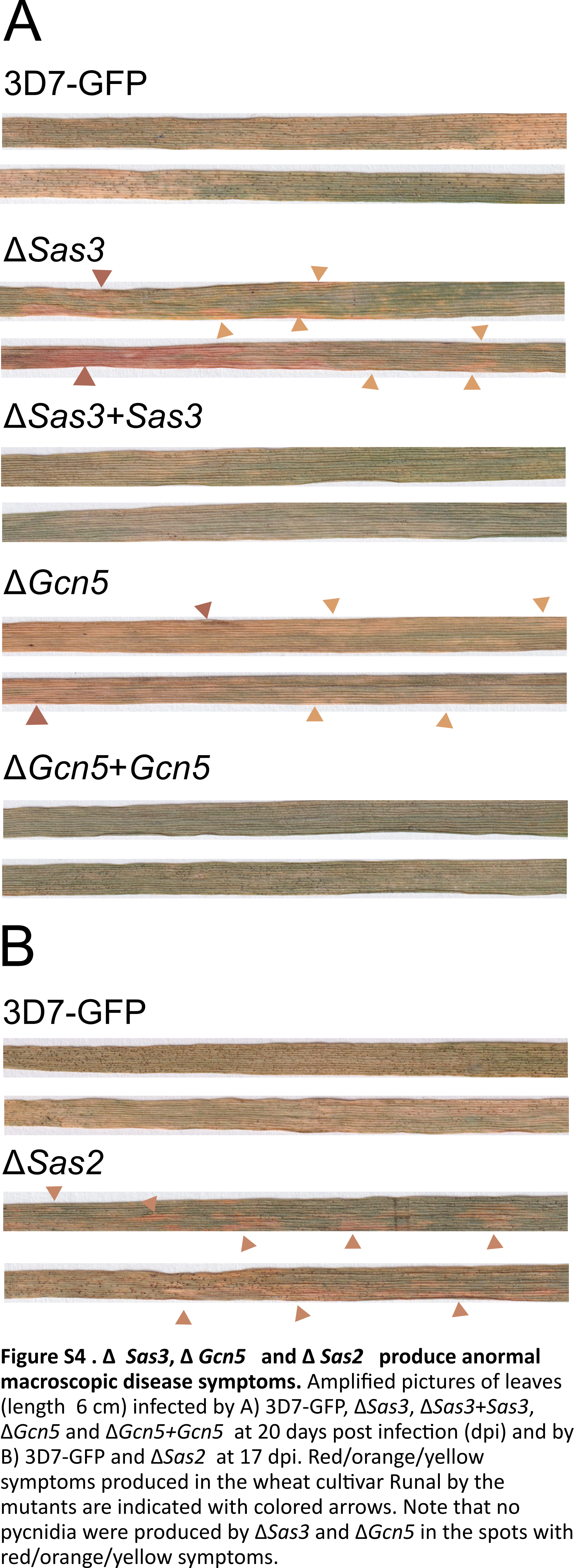

Supplement: Figure S4 — ∆Sas3, ∆Gcn5, and ∆Sas2 produce anormal macroscopic disease symptoms. [file mbio.01386-23-s0004.png]
