# Supplementary material for: Sas3-mediated histone acetylation regulates effector gene activation in a fungal plant pathogen
Source: mBio. 2023 Aug 29;14(5):e01386-23. doi: 10.1128/mbio.01386-23 (PMC10653901; doi:10.1128/mbio.01386-23)
Supplement: Figure S5 — Complementation of ΔSas3, ΔGcn5, and ΔElp3 recover the virulence phenotype. [file mbio.01386-23-s0005.pdf]

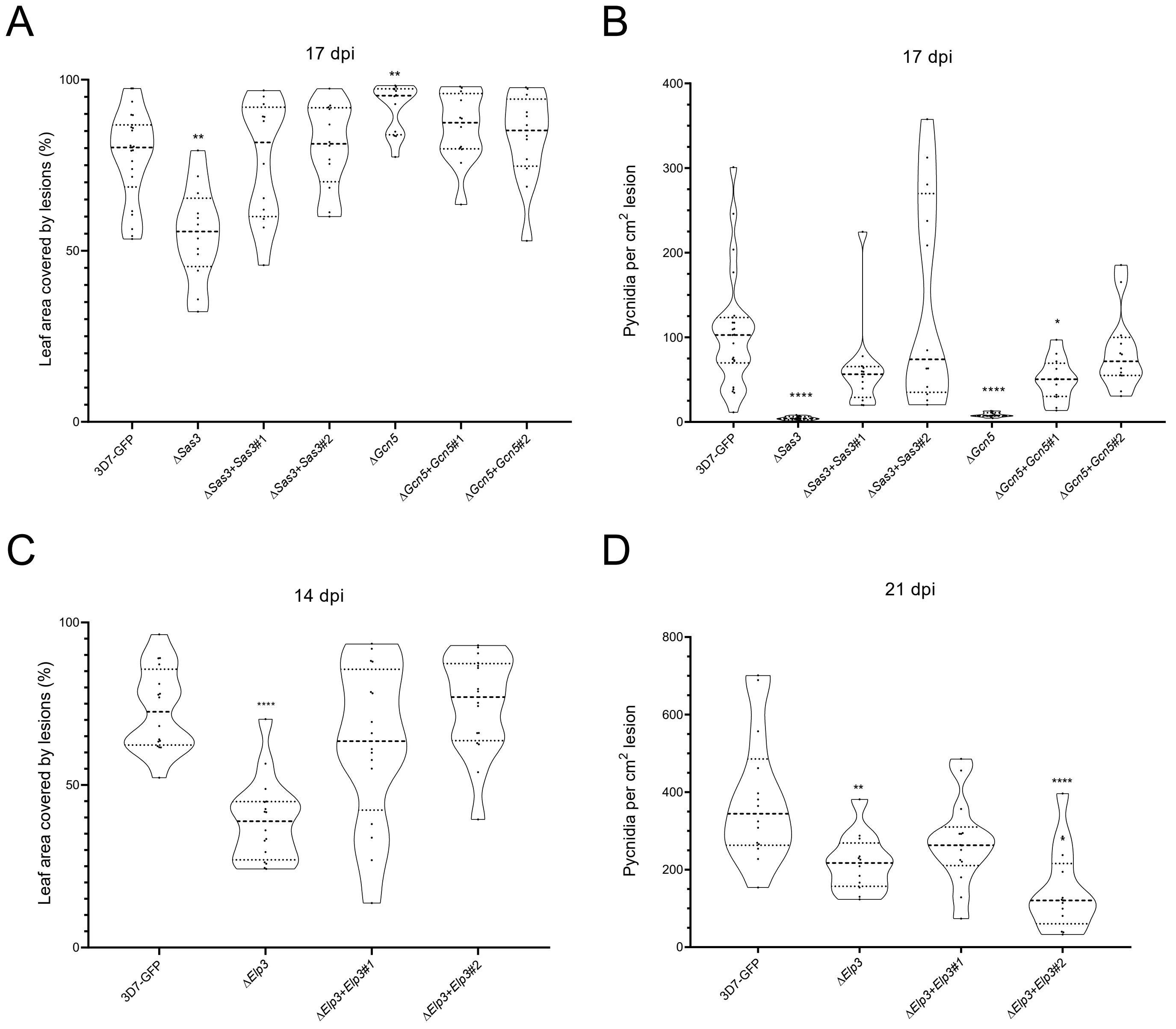

Figure S5. Complementation of Δ*Sas3*, Δ*Gcn5* and Δ*Elp3* recover the virulence phenotype. Percentage of leaf area covered by lesions (PLACL, A) and pycnidia per cm^2 of lesion (B) at 17 days post infection (dpi) in Δ*Sas3* and Δ*Gcn5* complementation lines of *Zymoseptoria tritici* infecting wheat plants of cultivar Runal. PLACL at 14 dpi (C) and pycnidia per cm^2 of lesion at 21 dpi (D) produced by Δ*Elp3* complementation lines. Dashed lines represent the median, dotted lines represent first, and third quartiles and black dots represent individual data points. Asterisks indicate statistically significant differences with 3D7-GFP according to the Kruskal-Wallis non-parametric statistical and posthoc uncorrected Dunn's tests (* $p < 0.05$; ** $p < 0.01$; **** $p < 0.0001$).
