# Supplementary material for: Sas3-mediated histone acetylation regulates effector gene activation in a fungal plant pathogen
Source: mBio. 2023 Aug 29;14(5):e01386-23. doi: 10.1128/mbio.01386-23 (PMC10653901; doi:10.1128/mbio.01386-23)
Supplement: Figure S6 — PCR screening strategy to identify gene deletion mutants. [file mbio.01386-23-s0006.pdf]

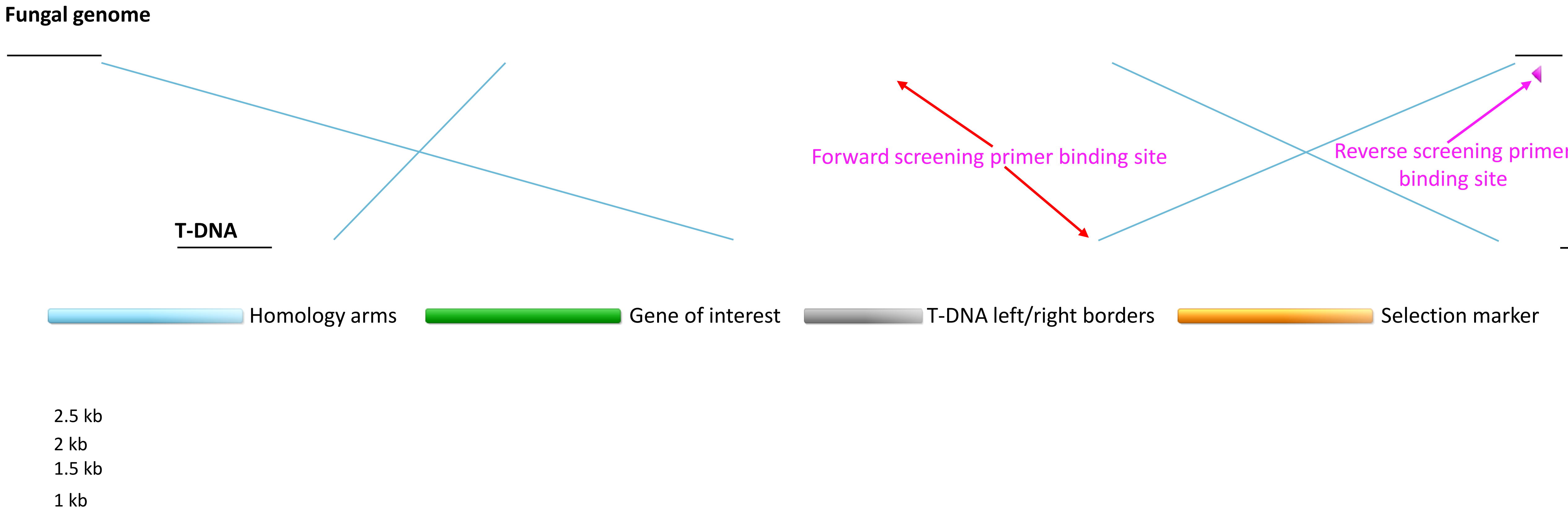

Figure S6. PCR screening strategy to identify gene deletion mutants. Top: Blue crosses represent homologous recombination events during fungal transformation. The forward screening primer binding sites within the gene of interest and the T-DNA are identical. Sizes of different parts do not correspond to a specific construct used in this study. Bottom: Example screening results for Δ*Elp3* mutant lines using liquid cultures directly added to the reaction. PCRs using forward and reverse screening primers yield distinct amplicons for native genes (1967 bp) and disrupted genes (1058 bp). Each lane represents a different mutant line.
