# Supplementary material for: Sas3-mediated histone acetylation regulates effector gene activation in a fungal plant pathogen
Source: mBio. 2023 Aug 29;14(5):e01386-23. doi: 10.1128/mbio.01386-23 (PMC10653901; doi:10.1128/mbio.01386-23)
Supplement: Table S1 — Five lysine acetyltransferases (KATs) from the MYST and GNAT families in Z. tritici were identified using BLASTp. [file mbio.01386-23-s0008.docx]

**Table S1. Five lysine acetyltransferases (KATs) from the MYST and GNAT families in Z. tritici were identified using BLASTp.** KATs of Saccharomyces cerevisiae, query cover, E-value, percentage of identity, accession number and gene ID of the best hit in Z. tritici are indicated.

| *S. cerevisiae* KAT | Query Cover | E- value | Percentage of Identity | Accession  (*Z. tritici*) | Associated *Z. tritici* gene ID |
| --- | --- | --- | --- | --- | --- |
| Gcn5 | 80% | 2e-164 | 60.76% | SMQ49624.1 | *3D7.g4775* |
| Elp3 | 99% | 0.0 | 74.24% | SMQ53347.1 | *3D7.g8500* |
| Sas2 | 60% | 1e-46 | 39.71% | SMQ51878.1 | *3D7.g7031* |
| Sas3 | 47% | 9e-79 | 36.83% | SMQ49112.1 | *3D7.4263* |
| Esa1 | 99% | 5e-162 | 49.41% | SMQ54127.1 | *3D7.9281* |
