# Supplementary material for: Sas3-mediated histone acetylation regulates effector gene activation in a fungal plant pathogen
Source: mBio. 2023 Aug 29;14(5):e01386-23. doi: 10.1128/mbio.01386-23 (PMC10653901; doi:10.1128/mbio.01386-23)
Supplement: Table S2 — NCBI accession numbers used for performing the phylogenetic tree. [file mbio.01386-23-s0009.docx]

**Table S2.** **NCBI accession numbers used for performing the phylogenetic tree.**

| KAT | Organism | NCBI accession number |
| --- | --- | --- |
| Ngs1 | *Neurospora crassa* | CAE85548.1 |
| Ngs1 | *Fusarium oxysporum* | KAF6521047.1 |
| Ngs1 | *Trichoderma reesei* | XP_006966911.1 |
| Ngs1 | *Aspergillus fumigatus* | KAH3100114.1 |
| Ngs1 | *Histoplasma capsulatum* | QSS62698.1 |
| Ngs1 | *Zymoseptoria tritici* | SMQ47703 |
| Ngs1 | *Candida albicans* | AOW30790.1 |
| Sas2 | *Saccharomyces cerevisiae* | DAA10024.1 |
| Sas2 | *Fusarium oxysporum* | SCO82216.1 |
| Sas2 | *Kluyveromyces marxianus* | XP_022675924 |
| Sas2 | *Beauveria bassiana* | KAF1731046.1 |
| Sas2 | *Zygosaccharomyces mellis* | GCE98240.1 |
| Sas2 | *Fusarium graminearum* | XP_011324667.1 |
| Sas2 | *Zymoseptoria tritici* | SMQ51878 .1 |
| Sas3 | *Saccharomyces cerevisiae* | DAA07067.1 |
| Sas3 | *Fusarium graminearum* | XP_011320283.1 |
| Sas3 | *Metarhizium robertsii* | XP_007818471.1 |
| Sas3 | *Fusarium oxysporum* | EWZ39814.1 |
| Sas3 | *Magnaphorte oryzae* | XP_003713627.1 |
| Sas3 | *Zymoseptoria tritici* | SMQ49112.1 |
| Esa1 | *Saccharomyces cerevisiae* | DAA11012.1 |
| Esa1 | *Magnaphorte oryzae* | XP_003719696.1 |
| Esa1 | *Fusarium graminearum* | Q4IEV4.1 |
| Esa1 | *Neurospora crassa* | XP_962217.1 |
| Esa1 | *Zymoseptoria tritici* | SMQ54127.1 |
| Gcn5 | *Saccharomyces cerevisiae* | DAA07067.1 |
| Gcn5 | *Ustylago maydis* | CAC80426.1 |
| Gcn5 | *Fusarium oxysporum* | EWZ52160.1 |
| Gcn5 | *Neurospora crassa* | XP_001728480.2 |
| Gcn5 | *Schizosaccharomyces pombe* | Q9UUK2.1 |
| Gcn5 | *Zymoseptoria tritici* | SMQ49624.1 |
| Elp3 | *Saccharomyces cerevisiae* | DAA11347.1 |
| Elp3 | *Fusarium graminearum* | XP_011317913.1 |
| Elp3 | *Magnaphorte oryzae* | XP_003710346.1 |
| Elp3 | *Schizosaccharomyces pombe* | NP_594862.1 |
| Elp3 | *Fusarium oxisporum* | XP_018239078.1 |
| Elp3 | *Candida albicans* | KAF6072097.1 |
| Elp3 | *Zymoseptoria tritici* | SMQ53347.1 |
