# Supplementary material for: Sas3-mediated histone acetylation regulates effector gene activation in a fungal plant pathogen
Source: mBio. 2023 Aug 29;14(5):e01386-23. doi: 10.1128/mbio.01386-23 (PMC10653901; doi:10.1128/mbio.01386-23)
Supplement: Table S3 — Primers used in this work. [file mbio.01386-23-s0010.docx]

**Table S3. Primers used in this work.**

| **Primer name** | **Primer sequence** | **Purpose** |
| --- | --- | --- |
| ASV18p_IF_KO-HAT_SAS2_F5 | TAATTAAGATATCGAGCTCGGTGAGATCCTCGTAGTAGTCGT | Construct-KO-HAT_SAS2 with ASV19p |
| ASV19p_IF_KO-HAT_SAS2_R5 | GGAGATGTGGAGTGGGGAGTGATTGCATTGAAACGG | Construct-KO-HAT_SAS2 with ASV18p |
| ASV20p_NAT_F | CCCACTCCACATCTCCACTC | Construct-KO-HAT_NAT Amplification with ASV21p |
| ASV21p_NAT_R | CCTCTTCGCTATTACGCCAG | Construct-KO-HAT_NAT Amplification with ASV20p |
| ASV22p_IF_KO-HAT_SAS2_F3 | CGTAATAGCGAAGAGGCATCTCACTCATCTCACTCTCCACCGACATTGTTTCCGACTG | Construct-KO-HAT_SAS2 with ASV23p |
| ASV23p_IF_KO-HAT_SAS2_R3 | CAGTGCCAAGCTTGCATGCCGTTTCCGTTCAACACAGCCT | Construct-KO-HAT_SAS2 with ASV22p |
| ASV24p_SAS2_ScreenP_F | CATCTCACTCATCTCACTCTC | Primer for screening KO-HAT_SAS2 with ASV25p |
| ASV25p_SAS2_ScreenP_R | GGATACGCAATGAACTTCTGG | Primer for screening KO-HAT_SAS2 with ASV24p |
| ASV26p_IF_KO-HAT_NGS1_F5 | GCCGAATTCGAGCTCGGGTGTGGTGGAAACTCTCCC | Construct KO-HAT_NGS1 with ASV27p |
| ASV27p_IF_KO-HAT_NGS1_R5 | GGAGATGTGGAGTGGGTTTGCATGGATTTGAGGAGGT | Construct KO-HAT_NGS1 with ASV26p |
| ASV28p_IF_KO-HAT_NGS1_F3 | CGTAATAGCGAAGAGGGATTCCTCACATTCGTCAACACCATCCTGCTGGCACTATTGG | Construct KO-HAT_NGS1 with ASV29p |
| ASV29p_IF_KO-HAT_NGS1_R3 | TAAAGCTTGCATGCCGCGATACTTCCTCACTACCC | Construct KO-HAT_NGS1 with ASV28p |
| ASV30p_KO-HAT_NGS1_ScreenP_F | GATTCCTCACATTCGTCAACAC | Primer for screening KO-HAT_NGS1 with ASV31p |
| ASV31p_KO-HAT_NGS1_ScreenP_R | ACTTCTTCTCGCTACCTCCTG | Primer for screening KO-HAT_NGS1 with ASV30p |
| ASV38p_KO-HAT_SAS3_F5 | GCCGAATTCGAGCTCGGGTCAAGGCGATGTATTTCC | Construct KO-HAT_SAS3 with ASV39p |
| ASV39p_KO-HAT_SAS3_R5 | GGAGATGTGGAGTGGGCATGTTGGTGGTTGAACTTGAG | Construct KO-HAT_SAS3 with ASV38p |
| ASV40p_KO-HAT_SAS3_F3 | CGTAATAGCGAAGAGGCTTGAAATCATGTGGCTCGTGGTTGTGCCTTGTAATTCACGCC | Construct KO-HAT_SAS3 with ASV41p |
| ASV41p_KO-HAT_SAS3_R3 | TAAAGCTTGCATGCCAATGCCTTGGTCGCTTTCCT | Construct KO-HAT_SAS3 with ASV40p |
| ASV42p_ScreenP_SAS3KO-HAT_F | CTTGAAATCATGTGGCTCGTGG | Primer for screening KO-HAT_SAS3 with ASV43p |
| ASV43p_ScreenP_SAS3KO-HAT_R | GCCTTCGTGTTGTCTGTCTG | Primer for screening KO-HAT_SAS3 with ASV42p |
| ASV44p_KO-HAT_GCN5_F5 | GCCGAATTCGAGCTCGGGAGGTGGAGTGTAGGTATAGG | Construct KO-HAT_GCN5 with ASV45p |
| ASV45p_KO-HAT_GCN5_R5 | GGAGATGTGGAGTGGGAGAGCGAGGTCAAGTTGTGAG | Construct KO-HAT_GCN5 with ASV44p |
| ASV46p_KO-HAT_GCN5_F3 | CGTAATAGCGAAGAGGAGTGAAGAAGCCTCCAGCAGGAATTACAAGCTTGGCTCAC | Construct KO-HAT_GCN5 with ASV47p |
| ASV47p_KO-HAT_GCN5_R3 | TAAAGCTTGCATGCCAATGCGCGGTATTGATTGAG | Construct KO-HAT_GCN5 with ASV46p |
| ASV48p_ScreenP_GCN5KO-HAT_F | AGTGAAGAAGCCTCCAGCAG | Primer for screening KO-HAT_GCN5 with ASV49p |
| ASV49p_ScreenP_GCN5KO-HAT_R | TATACCTCTCCTCGCCACTC | Primer for screening KO-HAT_GCN5 with ASV48p |
| ASV50p_KO-HAT_ELP3KO-HAT_F5 | GCCGAATTCGAGCTCGCGCCAAAGCAGTGATCAACG | Construct KO-HAT_ELP3 with ASV51p |
| ASV51p_KO-HAT_ELP3KO-HAT_R5 | GGAGATGTGGAGTGGGATTGAGCAATGCCGACTGTG | Construct KO-HAT_ELP3 with ASV50p |
| ASV52p_KO-HAT_ELP3KO-HAT_F3 | CGTAATAGCGAAGAGGGTGTGAGACGACCTTGAATCCAGGTCCTGGAGGTGTTGTAGC | Construct KO-HAT_ELP3 with ASV53p |
| ASV53p_KO-HAT_ELP3KO-HAT_R3 | TAAAGCTTGCATGCCGAAATTAGATGTAATCAAGCCCGC | Construct KO-HAT_ELP3 with ASV52p |
| ASV54p_ScreenP_ELP3KO-HAT_F | GTGTGAGACGACCTTGAATCCA | Primer for screening KO-HAT_ELP3 (ASV12) with ASV55p |
| ASV55p_ScreenP_ELP3KO-HAT_R | CCCTATTTGAGATTGCGTGTCAG | Primer for screening KO-HAT_ELP3 (ASV12) with ASV54p |
| ASVp158_Myc_R | AAATCGAATGTCCGCCTCGACTATAGGTCCTCTTCAGAAATAAGTTTT | Primer for construction of complementations with myc tag |
| ASVp165_Gcn5c_F | AATTAAGATATCGAGCTCGAAGGAGTAGGAGAATCTGGCG | Primer for construction of complementations of *Gcn5* |
| ASVp166_Gcn5_C_R | ACTTTTGTTCCTCAGGCTGCCGATTTGTCG | Primer for construction of complementations of *Gcn5* |
| ASVp167_Gcn5_Myc_F | GCAGCCTGAGGAACAAAAGTTGATCTCTGAAGAGG | Primer for construction of complementations of *Gcn5* + myc |
| ASVp193_SAS3_C_F | AATTAAGATATCGAGCTCGAAGGACGTACGGCGGTGCAG | Primer for construction of complementations of *Sas3* |
| ASVp194_SAS3_C_R | ACTTTTGTTCCTCATACTGGATCTCGTCATCCTCC | Primer for construction of complementations of *Sas3* |
| ASVp195_SAS3_myc_F | CCAGTATGAGGAACAAAAGTTGATCTCTGAAGAGG | Primer for construction of complementations of *Sas3* |
| ASVp204_SAS3_C_F2 | AATTAAGATATCGAGCTCGACCTTGGTCGCTTTCCTCCATTT | Primer for construction of complementations of *Sas3* |
| ASVp205_SAS3_C_F3 | AATTAAGATATCGAGCTCGAGCCTTCGTGTTGTCTGTCTG | Primer for construction of complementations of *Sas3* |
| ASVp206_SAS3_C_F4 | AATTAAGATATCGAGCTCGATCCAGCACGTACTCTGCTTATC | Primer for construction of complementations of *Sas3* |
| ASVp207_SAS3_C_R2 | AATTAAGATATCGAGCTCGATGGTTGAGGTGTTCGGAAGG | Primer for construction of complementations of *Sas3* |
| ASVp208_SAS3_C_R3 | AATTAAGATATCGAGCTCGAGCCGAAGAACTAGATCGTGGAG | Primer for construction of complementations of *Sas3* |
| ASVp219_AvrStb9_q1F | GCTTCGTGAGCGTGAATGAC | Primer for amplification of *AvrStb9* in qPCR |
| ASVp220_AvrStb9_q1R | GACGGATCGAGGTACCGAAC | Primer for amplification of *AvrStb9* in qPCR |
| ASVp221_AvrStb9_q2F | CTCGTATTTGCTTCCTCCGC | Primer for amplification of *AvrStb9* in qPCR |
| ASVp222_AvrStb9_q2R | ATCAAGTCCAAGGTGTCGGT | Primer for amplification of *AvrStb9* in qPCR |
| ASVp225_LM160_TFIIIC_qF1 | AGAGGGGTCCGTTCATCTCA | *TFIIIC* - Reference gene for ChIP-qPCR |
| ASVp226_LM161_TFIIIC_qR1 | GTCGAAGCAGTAGAGGCGTT | *TFIIIC* - Reference gene for ChIP-qPCR |
| ASVp232_H3_F1 | TCGCAAGTCCGCACCATCCA | *Histone H3* - Housekeeping gene for qPCR |
| ASVp232_H3_F1 | TCGCAAGTCCGCACCATCCA | *Histone H3* - Housekeeping gene for qPCR |
| ASVp238_B-Tub_F2 | GAGGAGTTCCCCGACCGCAT | *Β-tubulin* - Housekeeping gene for qPCR |
| ASVp239_B-Tub_R2 | AGCTGGTGGACGGAGAGGGT | *Β-tubulin* - Housekeeping gene for qPCR |
| ASVp242_AvrStb6_1000_ChIPqF | TCCCTGTCCGGAAACTAGGA | Primer for amplification of 1000 bp upstream of *AvrStb6* in ChIP-qPCR |
| ASVp243_AvrStb6_1000_ChIPqR | GGGCCTGCTTAATAAATGGCG | Primer for amplification of 1000 bp upstream of *AvrStb6* in ChIP-qPCR |
| ASVp246_AvrStb6_500_ChIPqF | TTTCCGGCACTTGCCTAACT | Primer for amplification of 500 bp upstream of *AvrStb6* in ChIP-qPCR |
| ASVp247_AvrStb6_500_ChIPqR | TCCGCGCTATTCCTGTATGC | Primer for amplification of 500 bp upstream of *AvrStb6* in ChIP-qPCR |
| ASVp250_AvrStb6_300_ChIPqF | CCAGGGGCTATGCACTACTT | Primer for amplification of 300 bp upstream of *AvrStb6* in ChIP-qPCR |
| ASVp251_AvrStb6_300_ChIPqR | CGGCTCCTGCACCCAAAATA | Primer for amplification of 300 bp upstream of *AvrStb6* in ChIP-qPCR |
| ASVp252_AvrStb6_50_ChIPqF | CTCAACCAAGACCAAAGCAGC | Primer for amplification of 50 bp upstream of *AvrStb6* in ChIP-qPCR |
| ASVp253_AvrStb6_50_ChIPqR | AATGGATTCGGCGACAGGTG | Primer for amplification of 50 bp upstream of *AvrStb6* in ChIP-qPCR |
| ASVp256_AvrStb6_ORF_ChIPqF | ATAGATCTCTGCAAGGCGGG | Primer for amplification of ORF of *AvrStb6* in ChIP-qPCR |
| ASVp257_AvrStb6_ORF_ChIPqR | ACACCTTGGATATTGCCCGT | Primer for amplification of ORF of *AvrStb6* in ChIP-qPCR |
| ASVp258_1qPCR_AvrStb6_fw (LM254) | AAGGCGGGTCCTAGTTGCT | Primer for amplification of *AvrStb6* in qPCR |
| ASVp259_1qPCR_AvrStb6_rv (LM255) | AAGCTGCTGTGATGGAGAGC | Primer for amplification of *AvrStb6* in qPCR |
| LM170_581_qF2 | AGCATTCGACGACTGTTGGT | Primer for *Avr3D1* amplification with LM171 in ChIP-qPCR |
| LM171_581_qR2 | GGTGGCTAGCTTGGAACTGT | Primer for *Avr3D1* amplification with LM170 in ChIP-qPCR |
| LM288_Cellulase_qF4 | AACCAATACGGCGTCCAGA | Primer for *Mycgr3G76589* qPCR amplification with LM289 |
| LM289_Cellulase_qR4 | CCACTCCTGCTCACCAAGTC | Primer for *Mycgr3G76589* qPCR amplification with LM288 |
| LM343B_NATqF | AGGTCACCAACGTCAACG | Primer for Copy number of resistance cassette (NatR) with LM344B |
| LM344B_NATqR | CTCATGTAGAGCGCCAGC | Primer for Copy number of resistance cassette (NatR) with LM343B |
| CDC48_QPCR_F2 | GTCCTCCTGGCTGTGGTAAAAC | Primer for amplification of cell division control 48 gene in wheat |
| CDC48_QPCR_R2 | AGCAGCTCAGGTCCCTTGATAC | Primer for amplification of cell division control 48 gene in wheat |
